# Supplementary material for: SynthCraft: An AI partner for synthetic data generation to support data access and augmentation in healthcare
Source: PLOS Digit Health. 2026 Mar 9;5(3):e0001290. doi: 10.1371/journal.pdig.0001290 (PMC12970901; doi:10.1371/journal.pdig.0001290)
Supplement: S1 Text — Table A. Summary of synthetic data generator tools available in SynthCraft. Table B. Data quality metrics in SynthCraft. Table C. Performance metrics for generated synthetic datasets (NHANES). Table D. Discrimination (AUC) by sub-group for logistic regression models trained on the real data and on synthetic data (NHANES dataset). Table E. Discrimination (AUC) by sub-group for logistic regression models trained on the real data and on real data augmented with synthetic data (NHANES dataset). Table F. Exhaustive list of performance metrics for generated synthetic datasets (TCGA dataset). Table G. Comparison of variable distributions in the real and synthetically generated datasets (TCGA purity dataset). Table H. Performance on the TCGA gene purity dataset. Table I. Ablation studies comparing SynthCraft against GPT-4o. Fig A. Workflow for the NHANES dataset. (PDF) [file pdig.0001290.s001.pdf]

SUPPLEMENT TO: Callender T, Boyd A, Davis R, Rurhberg Estevez S, Ferres JR, van der Schaar, M. SynthCraft: an AI partner for synthetic data generation to support data access and augmentation in healthcare

TABLE OF CONTENTS

SUPPLEMENTARY METHODS 2

Synthcity's in-built four-stage augmentation pipeline 2

Report generated on completing synthetic data generation for the NHANES dataset 3

Report generated on completing synthetic data generation for the TCGA dataset 3

SUPPLEMENTARY TABLES 5

Table A. Summary of synthetic data generator tools available in SynthCraft 5

Table B. Data quality metrics in SynthCraft 7

Table C. Performance metrics for generated synthetic datasets (NHANES) 9

Table D. Discrimination (AUC) by sub-group for logistic regression models trained on the real data and on synthetic data (NHANES dataset) 11

Table E. Discrimination (AUC) by sub-group for logistic regression models trained on the real data and on real data augmented with synthetic data (NHANES dataset) 13

Table F. Exhaustive list of performance metrics for generated synthetic datasets (TCGA dataset) 15

Table G. Comparison of variable distributions in the real and synthetically generated datasets (TCGA purity dataset) 17

Table H. Performance on the TCGA gene purity dataset 18

Table I. Ablation studies comparing SynthCraft against GPT-4o 19

SUPPLEMENTARY FIGURES 21

Fig A. Workflow for the NHANES dataset 21

REFERENCES 29

SUPPLEMENTARY METHODS

Synthcity's in-built four-stage augmentation pipeline

Below are the four stages summarizing the synthetic data augmentation pipeline used to address class imbalances in ethnicity for the NHANES data.

Stage 1: Baseline enumeration
First the number of real participants in each self-reported ethnic subgroup are calculated. This audit revealed that several minority groups were markedly under-represented relative to the largest (majority) subgroup.

Stage 2: Enrichment target calculation
For each under-represented ethnicity, we computed an exact augmentation target - the number of additional records required to bring that subgroup's total up to parity with the majority group. This ensured that, once augmented, every ethnicity would appear equally often in downstream model training.

Stage 3: Conditional synthetic generation
We trained the synthetic generators on the full suite of NHANES variables. At synthesis time, the model was conditioned explicitly on the ethnicity label and the generator produced the requisite number of new records for each subgroup to better balance the ethnicity distribution in the dataset. Although entirely artificial, these samples preserved the multivariate relationships present in the original data.

Stage 4: Cohort integration and evaluation
Finally, the synthetic records were appended to the original NHANES data, yielding a balanced training set in which all ethnicities were equally represented. When classifiers and regression models were trained on this augmented cohort.

Report generated on completing synthetic data generation for the NHANES dataset

Let's summarize what has been done in the project so far:

1. **Upload Data File**:
    - The dataset nhanes_simple_v2.csv was successfully uploaded.
2. **Experiment Setup and Research Question**:
    - The user defined the research question and confirmed the classification task with the target column mcq160e.
3. **Exclude/Keep Columns**:
    - The column seqn was excluded from the analysis.
4. **Perform EDA**:
    - Exploratory Data Analysis (EDA) was performed on the dataset, revealing various statistical insights, correlations, and outliers.
5. **Generate Descriptive Statistics**:
    - Descriptive statistics were generated for the dataset.
6. **Confirm ML Problem Type**:
    - The ML problem type was confirmed to be classification.
7. **Select Synthetic Data Generation Method**:
    - The user chose the following synthetic data generators: CTGAN, TVAE, DDPM, ADS-GAN, and PATE-GAN.
8. **Generate Synthetic Data**:
    - Synthetic data was generated and augmented with the fairness column ridreth1.
9. **Perform EDA on Synthetic Data**:
    - EDA was performed on the synthetic dataset pategan_synthetic_data.csv.
10. **Generate Descriptive Statistics for Synthetic Data**:
    - Descriptive statistics were generated for the synthetic dataset.
11. **Plot Synthetic Data**:
    - Synthetic data was plotted for all selected methods, and the plots were saved in the working directory.

Report generated on completing synthetic data generation for the TCGA dataset

Let's summarize what has been done in the project so far:

1. **Upload Data File:**
    - The dataset named cleaned_gene_purity_data_v2.csv was uploaded.
2. **Experiment Setup and Research Question:**
    - The research problem was identified as a regression problem with the target column being "purity".
3. **Exclude/Keep Columns:**
    - The column "Unnamed: 0" was excluded from the dataset.

4. **Perform EDA:**
    - Exploratory Data Analysis (EDA) was performed on the dataset, revealing various statistics and correlations.
5. **Generate Descriptive Statistics:**
    - Descriptive statistics were generated for the dataset.
6. **Confirm ML Problem Type:**
    - The problem type was confirmed to be regression.
7. **Select Synthetic Data Generation Method:**
    - The user selected the following synthetic data generation methods: CTGAN, TVAE, DDPM, ADS-GAN, and PATE-GAN.
8. **Generate Synthetic Data:**
    - Synthetic data was generated successfully using the selected methods.
9. **Perform EDA on Synthetic Data:**
    - EDA was performed on the PATE-GAN synthetic dataset.
10. **Generate Descriptive Statistics for Synthetic Data:**
    - Descriptive statistics were generated for the PATE-GAN synthetic dataset.
11. **Plot Synthetic Data:**
    - Synthetic data plots were generated for all selected methods and saved in the working directory. diredirectory.

SUPPLEMENTARY TABLES

Table A. Summary of synthetic data generator tools available in SynthCraft

Generator	Description
**A) General purpose generators**	
Dummy sampler	Generates synthetic data by randomly sampling from real data.
Uniform sampler	Generates data by uniformly sampling across the variable range.
Marginal distributions	Samples each feature independently based on its marginal distribution.
CTGAN	GAN-based synthesizer tailored to tabular data with mixed types.
TVAE	Variational autoencoder adapted for tabular data.
Normalising flows	Neural normalizing flows for complex distribution modelling.
RTVAE	Recurrent VAE for longitudinal/time-series data.
Bayesian networks	Learns probabilistic graphical models from data.
GREAT	GAN with reinforcement learning-based generator optimization.
DDPM	Denoising diffusion probabilistic model for high-quality synthetic generation.
ARF	Adversarial random forest synthesizer using decision tree ensembles.
**B) Privacy-preserving generators**	
ADS-GAN	Adversarially trains to fool re-identification attacks.
AIM	Minimizes mutual information between synthetic and real data.
PATE-GAN	Differentially private GAN using the PATE framework.
DECAF	Privacy-preserving framework using attribute conditioning.
PrivBayes	Bayesian network generator with differential privacy guarantees.
DPGAN	GAN with gradient-level differential

Summary of the general-purpose synthetic data generation methods (**A**) available in and privacy-preserving data generation methods (**B**) integrated into SynthCraft. The general-purpose generators include a range of statistical, adversarial, and deep generative models capable of modelling complex data distributions across both cross-sectional and temporal modalities. The privacy-preserving methods incorporate formal privacy mechanisms, such as differential privacy, mutual information minimization, and adversarial defence to reduce the risk of re-identification while maintaining utility in synthetic data.

Table B. Data quality metrics in SynthCraft

Metric	Description
**A) Foundational aspects**	
Data mismatch	Measures structural incompatibility between real and synthetic datasets.
Common rows proportion	Proportion of synthetic rows identical to real rows (potential leakage).
Nearest synthetic neighbour distance	Distance from real samples to their closest synthetic neighbours.
Close values probability	Probability of synthetic samples closely matching real ones.
Distant values probability	Probability that synthetic data are unreasonably far from real samples.
**B) Statistical Similarity**	
Jensen-Shannon distance	Measures similarity between distributions via Jensen-Shannon divergence.
Chi-squared test	Compares categorical distributions using Chi-squared test.
Feature Corelation	Measures correlation preservation across features.
Kullback-Leibler divergence	KL divergence from synthetic to real (inverted).
Kolmogorov-Smirnov test	Kolmogorov-Smirnov test for univariate distribution similarity.
Empirical maximum mean discrepancy	Measures distributional distance using kernel-based two-sample test.
Wasserstein distance	Earth-mover's distance between real and synthetic samples.
Precision, Recall, Density & Coverage statistics	Precision/Recall metrics for distribution comparison using nearest neighbours.
Alpha precision (alpha precision, beta-recall, authenticity)	A 3-dimensional score of synthetic data quality consisting of measures of synthetic data fidelity (alpha-precision), diversity (beta-recall), and generalisability (authenticity) [1].
Kaplan-Meier distance	Distance between real and synthetic Kaplan-Meier curves (for survival data).
**C) Downstream Performance**<br><br>Models are trained (linear regression, multi-layer perceptron [MLP], or XGBoost) on both real and synthetic data before they are evaluated on the real test data. R^2 is used for regression and the area under the receiver operating curve (AUC) for classification.	
**D) Privacy Risk Assessment**	
Delta presence	Measures the likelihood a real record exists in the synthetic set.
k-anonymization	Checks how many real samples are indistinguishable from others.
k-map	Assesses similarity of synthetic data to known real data populations.
Distinct l-diversity	Measures diversity of sensitive attributes within anonymized groups.
Identifiability score	Quantifies re-identification risk for individual records.

Description of quality metrics implemented in SynthCraft; adapted from Synthcity[2], available at: https://github.com/vanderschaarlab/synthcity. Foundational aspects of synthetic data quality (**A**) related to those assessing data leakage, sample similarity, and basic distributional plausibility between real and synthetic datasets. The statistical similarity metrics **(B)** assess univariate and multivariate distributional alignment, dependency structure preservation, and overall fidelity of the synthetic data to the real data distribution. The downstream performance evaluation **(C)** metrics quantifies the utility of synthetic data. Models trained on synthetic data are compared to those trained on real data using various predictive algorithms, providing insight into the practical value of the generated datasets for real-world machine learning tasks. The privacy risk assessment metrics **(D)** assess individual-level disclosure risks, including re-identification, attribute inference, and sample uniqueness. Together, they provide a rigorous and interpretable evaluation of the privacy protections afforded by synthetic datasets.

Table C. Performance metrics for generated synthetic datasets (NHANES)

Performance metric	Synthetic datasets			
	ADS-GAN	CTGAN	DDPM	PATE-GAN
Data mismatch ↓	0.0±0.0	0.0±0.0	0.0±0.0	0.0±0.0
Common rows proportion ↓	0.0±0.0	0.0±0.0	0.0±0.0	0.0±0.0
Nearest synthetic neighbour distance ↑	0.122±0.014	0.096±0.025	0.177±0.009	0.174±0.024
Close values probability ↑	0.884±0.031	0.924±0.049	0.659±0.034	0.693±0.104
Distant values probability ↓	0.005±0.002	0.002±0.001	0.002±0.001	0.003±0.0
Jensen-Shannon distance ↓	0.011±0.001	0.011±0.001	0.007±0.001	0.025±0.006
Chi-squared test ↑	0.422±0.049	0.358±0.042	0.809±0.071	0.716±0.047
Kullback-Leibler divergence ↑	0.84±0.018	0.809±0.026	0.954±0.004	0.793±0.081
Kolmogorov-Smirnov test ↑	0.888±0.004	0.876±0.017	0.946±0.008	0.722±0.072
Empirical maximum mean discrepancy ↓	0.003±0.001	0.003±0.001	0.003±0.001	0.003±0.001
Wasserstein distance ↓	0.038±0.004	0.043±0.008	0.159±0.006	0.191±0.039
Precision ↑	0.86±0.028	0.851±0.027	0.953±0.007	0.199±0.059
Recall ↑	0.912±0.026	0.928±0.014	0.983±0.002	0.842±0.073
Density ↑	0.675±0.061	0.636±0.025	0.971±0.025	0.092±0.023
Coverage ↑	0.811±0.046	0.789±0.045	0.964±0.006	0.207±0.047
Alpha precision (one-class) ↑	0.466±0.016	0.431±0.02	0.924±0.02	0.152±0.009
Beta-recall (one-class) ↑	0.292±0.024	0.25±0.006	0.442±0.015	0.077±0.003
Authenticity (one-class) ↑	0.649±0.026	0.643±0.025	0.51±0.02	0.915±0.032
(Performance) Linear model (train real test real) ↑	0.888±0.0	0.888±0.0	0.82±0.0	0.888±0.0
(Performance) Linear model (train synthetic test synthetic) ↑	0.858±0.019	0.844±0.02	0.688±0.039	0.586±0.101
(Performance) Linear model (train synthetic test real) ↑	0.835±0.039	0.78±0.041	0.655±0.115	0.62±0.046
(Performance) MLP (train real test real) ↑	0.5±0.0	0.5±0.0	0.5±0.0	0.5±0.0
(Performance) MLP (train synthetic test synthetic) ↑	0.5±0.0	0.5±0.0	0.5±0.0	0.5±0.0
(Performance) MLP (train synthetic test real) ↑	0.5±0.0	0.5±0.0	0.5±0.0	0.5±0.0
(Performance) XGBoost (train real test real) ↑	0.773±0.0	0.773±0.0	0.8±0.0	0.773±0.0
(Performance) XGBoost (train synthetic test synthetic) ↑	0.743±0.04	0.718±0.051	0.704±0.059	0.548±0.071

Table C. Performance metrics for generated synthetic datasets (NHANES)

Performance metric	Synthetic datasets			
	ADS-GAN	CTGAN	DDPM	PATE-GAN
(Performance) XGBoost (train synthetic test real) ↑	0.692±0.046	0.661±0.145	0.646±0.121	0.463±0.12
(Performance) Linear model (train augmented test augmented) ↑	0.861±0.001	0.861±0.001	0.822±0.0	0.861±0.001
(Performance) Linear model (train augmented test real) ↑	0.928±0.002	0.918±0.005	0.808±0.019	0.779±0.065
(Performance) MLP (train augmented test augmented) ↑	0.5±0.0	0.5±0.0	0.5±0.0	0.5±0.0
(Performance) MLP (train augmented test real) ↑	0.5±0.0	0.5±0.0	0.5±0.0	0.5±0.0
(Performance) XGBoost (train augmented test augmented) ↑	0.718±0.0	0.718±0.0	0.743±0.001	0.718±0.0
(Performance) XGBoost (train augmented test real) ↑	0.913±0.023	0.906±0.028	0.941±0.015	0.89±0.063
Delta presence ↓	11.167±3.52	7.292±4.151	1.747±0.285	46.667±40.086
k-anonymization (real data) ↑	3.0±0.0	3.0±0.0	28.0±0.0	3.0±0.0
k-anonymization (synthetic data) ↑	3.667±3.091	2.333±1.886	20.667±8.34	1.333±0.471
K-map ↑	1.667±0.471	1.0±0.0	23.667±0.943	1.333±0.471
Distinct l-diversity (real data) ↑	3.0±0.0	3.0±0.0	28.0±0.0	3.0±0.0
Distinct l-diversity (synthetic data) ↑	3.667±3.091	2.333±1.886	20.667±8.34	1.333±0.471
Identifiability score ↓	0.287±0.018	0.259±0.004	0.495±0.021	0.042±0.013
Identifiability score (one-class) ↓	0.32±0.025	0.272±0.005	0.466±0.012	0.084±0.004

An exhaustive list of statistical and performance metrics for the synthetic versions of NHANES by generator. Arrows refer to whether a higher or a lower score is desirable. Refer to Supplementary Table 2 or the Synthcity library in Github (https://github.com/vanderschaarlab/synthcity/) for further details. Abbreviations: ADS-GAN, Anonymization through Data Synthesis using Generative Adversarial Networks; CTGAN, conditional table generative adversarial network; DDPM, denoising diffusion probabilistic models; MLP, multilayer perceptron; NHANES, National Health and Nutrition Examination Survey; PATE-GAN, Private Aggregation of Teacher Ensembles Generative Adversarial Networks; XGBoost, Extreme Gradient Boosting.

Table D. Discrimination (AUC) by sub-group for logistic regression models trained on the real data and on synthetic data (NHANES dataset)

		Synthetic datasets			
	**Real**	**ADS-GAN**	**CTGAN**	**DDPM**	**PATE-GAN**
Overall	0.818 (0.773-0.859)	0.781 (0.733-0.827)	0.797 (0.746-0.847)	0.747 (0.701-0.785)	0.620 (0.575-0.661)
Age (per year)					
<60	0.681 (0.524-0.825)	0.597 (0.437-0.751)	0.587 (0.420-0.749)	0.655 (0.516-0.782)	0.716 (0.624-0.799)
60-69	0.835 (0.777-0.888)	0.787 (0.722-0.851)	0.828 (0.768-0.882)	0.630 (0.545-0.712)	0.409 (0.308-0.511)
70-80	0.720 (0.651-0.786)	0.699 (0.627-0.768)	0.723 (0.654-0.793)	0.585 (0.505-0.665)	0.437 (0.359-0.516)
Gender					
Female	0.802 (0.748-0.852)	0.775 (0.719-0.824)	0.797 (0.741-0.850)	0.738 (0.691-0.785)	0.641 (0.589-0.700)
Male	0.814 (0.740-0.876)	0.749 (0.660-0.827)	0.764 (0.667-0.846)	0.732 (0.651-0.806)	0.649 (0.581-0.717)
Ethnicity*					
Other Hispanic	0.843 (0.732-0.933)	0.808 (0.692-0.908)	0.818 (0.681-0.929)	0.830 (0.732-0.907)	0.603 (0.507-0.695)
Non-Hispanic White	0.810 (0.758-0.860)	0.788 (0.732-0.844)	0.798 (0.740-0.857)	0.711 (0.659-0.762)	0.615 (0.554-0.670)
Non-Hispanic Black	0.871 (0.784-0.946)	0.790 (0.641-0.913)	0.828 (0.703-0.936)	0.825 (0.694-0.920)	0.578 (0.426-0.730)
Other Ethnicity	0.807 (0.678-0.920)	0.769 (0.605-0.898)	0.740 (0.574-0.892)	0.768 (0.636-0.897)	0.745 (0.629-0.842)
Body Mass Index (per kg/m^2)					
≤24	0.910 (0.850-0.960)	0.865 (0.776-0.936)	0.889 (0.802-0.957)	0.879 (0.811-0.941)	0.675 (0.598-0.750)
25-29	0.814 (0.748-0.875)	0.808 (0.735-0.873)	0.795 (0.715-0.865)	0.765 (0.711-0.816)	0.614 (0.552-0.674)
≥30	0.785 (0.717-0.850)	0.723 (0.645-0.796)	0.764 (0.692-0.833)	0.749 (0.680-0.816)	0.607 (0.538-0.684)

All models were trained to predict self-reported myocardial infarction on the respective dataset (shown by the column) and tested on the real data. 95% confidence intervals are percentiles of 1,000 bootstrap resamples.

*Within the Mexican American ethnicity sub-group there were fewer than 5 instances of self-reported myocardial infarction so AUC values were not derived.

Abbreviations: AUC, Area under the curve; ADS-GAN, Anonymization through Data Synthesis using Generative Adversarial Networks; CTGAN, conditional table generative adversarial network; DDPM, denoising diffusion probabilistic models; NHANES, National Health and Nutrition Examination Survey; PATE-GAN, Private Aggregation of Teacher Ensembles Generative Adversarial Networks.

Table E. Discrimination (AUC) by sub-group for logistic regression models trained on the real data and on real data augmented with synthetic data (NHANES dataset)

		Augmented datasets			
	Real	ADS-GAN	CTGAN	DDPM	PATE-GAN
Overall	0.818 (0.773-0.859)	0.810 (0.763-0.853)	0.813 (0.770-0.853)	0.799 (0.754-0.840)	0.793 (0.750-0.832)
Age (per year)					
<60	0.681 (0.524-0.825)	0.657 (0.506-0.793)	0.696 (0.554-0.817)	0.664 (0.538-0.781)	0.702 (0.579-0.812)
60-69	0.835 (0.777-0.888)	0.832 (0.776-0.886)	0.828 (0.768-0.882)	0.799 (0.736-0.859)	0.805 (0.740-0.867)
70-80	0.720 (0.651-0.786)	0.704 (0.634-0.770)	0.696 (0.628-0.761)	0.686 (0.618-0.750)	0.660 (0.585-0.734)
Gender					
Female	0.802 (0.748-0.852)	0.803 (0.748-0.853)	0.799 (0.747-0.847)	0.788 (0.737-0.834)	0.785 (0.732-0.832)
Male	0.814 (0.740-0.876)	0.810 (0.733-0.875)	0.815 (0.744-0.873)	0.779 (0.705-0.844)	0.791 (0.719-0.853)
Ethnicity*					
Other Hispanic	0.843 (0.732-0.933)	0.833 (0.716-0.923)	0.835 (0.725-0.921)	0.834 (0.733-0.917)	0.801 (0.696-0.892)
Non-Hispanic White	0.810 (0.758-0.860)	0.805 (0.753-0.856)	0.805 (0.755-0.854)	0.783 (0.729-0.835)	0.798 (0.750-0.845)
Non-Hispanic Black	0.871 (0.784-0.946)	0.862 (0.779-0.939)	0.866 (0.791-0.938)	0.858 (0.779-0.933)	0.815 (0.694-0.922)
Other Ethnicity	0.807 (0.678-0.920)	0.787 (0.631-0.917)	0.801 (0.656-0.923)	0.792 (0.652-0.919)	0.789 (0.623-0.920)
Body Mass Index (per kg/m^2)					
≤24	0.910 (0.850-0.960)	0.893 (0.827-0.948)	0.893 (0.829-0.943)	0.880 (0.813-0.934)	0.862 (0.789-0.923)
25-29	0.814 (0.748-0.875)	0.805 (0.734-0.865)	0.810 (0.747-0.865)	0.810 (0.752-0.861)	0.790 (0.725-0.849)
≥30	0.785 (0.717-0.850)	0.782 (0.714-0.845)	0.782 (0.714-0.847)	0.759 (0.688-0.825)	0.771 (0.703-0.835)

All models were trained to predict self-reported myocardial infarction on the respective dataset (shown by the column) and tested on the original real data. 95% confidence intervals are percentiles of 1,000 bootstrap resamples. For clarity, an augmented dataset includes the real data which has been combined with additional synthetic data.

*Within the Mexican American ethnicity sub-group there were fewer than 5 instances of self-reported myocardial infarction so AUC values were not derived.

Abbreviations: AUC, Area under the curve; ADS-GAN, Anonymization through Data Synthesis using Generative Adversarial Networks; CTGAN, conditional table generative adversarial network; DDPM, denoising diffusion probabilistic models; NHANES, National Health and Nutrition Examination Survey; PATE-GAN, Private Aggregation of Teacher Ensembles Generative Adversarial Networks.

Table F. Exhaustive list of performance metrics for generated synthetic datasets (TCGA dataset)

Performance metric	Synthetic datasets			
	ADS-GAN	CTGAN	DDPM	PATE-GAN
Data mismatch ↓	0.0±0.0	0.0±0.0	0.0±0.0	0.0±0.0
Common rows proportion ↓	0.0±0.0	0.0±0.0	0.0±0.0	0.0±0.0
Nearest synthetic neighbour distance ↑	0.009±0.001	0.01±0.001	0.021±0.009	0.021±0.0
Close values probability ↑	0.997±0.001	0.996±0.002	0.99±0.005	0.994±0.001
Distant values probability ↓	0.001±0.0	0.001±0.001	0.001±0.001	0.001±0.0
Jensen-Shannon distance ↓	0.004±0.001	0.002±0.001	0.001±0.001	0.005±0.0
Chi-squared test ↑	0.182±0.074	0.091±0.074	0.182±0.001	0.727±0.0
Kullback-Leibler divergence ↑	0.942±0.001	0.941±0.012	0.957±0.003	0.966±0.0
Kolmogorov-Smirnov test ↑	0.859±0.011	0.883±0.016	0.969±0.005	0.821±0.0
Empirical maximum mean discrepancy ↓	0.001±0.001	0.001±0.001	0.001±0.001	0.001±0.0
Wasserstein distance ↓	0.008±0.001	0.008±0.001	0.02±0.011	0.064±0.0
Precision ↑	0.953±0.003	0.961±0.006	0.97±0.003	0.804±0.0
Recall ↑	0.917±0.015	0.93±0.019	0.967±0.004	0.986±0.0
Density ↑	0.929±0.015	0.992±0.03	1.024±0.008	0.504±0.0
Coverage ↑	0.816±0.039	0.864±0.021	0.97±0.005	0.62±0.0
Alpha precision (one-class) ↑	0.843±0.02	0.819±0.008	0.897±0.008	0.69±0.0
Beta-recall (one-class) ↑	0.438±0.008	0.434±0.014	0.496±0.008	0.352±0.001
Authenticity (one-class) ↑	0.518±0.008	0.51±0.027	0.495±0.003	0.56±0.0
(Performance) Linear model (real data) r^2 ↑	0.222±0.0	0.222±0.0	0.222±0.0	0.222±0.0
(Performance) Linear model (synthetic data) r^2 ↑	0.099±0.018	0.086±0.082	0.217±0.006	0.001±0.0
(Performance) Linear model (train synthetic test real) r^2 ↑	0.285±0.059	0.303±0.019	0.292±0.009	0.001±0.0
(Performance) MLP (train real test real) r^2 ↑	0.001±0.0	0.001±0.0	0.001±0.0	0.001±0.0
(Performance) MLP	0.001±2204.678	0.001±11654.993	0.001±8375.526	0.001±0.0

Table F. Exhaustive list of performance metrics for generated synthetic datasets (TCGA dataset)

Performance metric	Synthetic datasets			
	ADS-GAN	CTGAN	DDPM	PATE-GAN
(train synthetic test synthetic) r^2↑				
(Performance) MLP (train synthetic test real) r^2↑	0.001±2780.465	0.001±17160.387	0.001±9712.445	0.001±0.001
(Performance) XGBoost (train real test real) r^2↑	0.402±0.0	0.402±0.0	0.402±0.0	0.402±0.0
(Performance) XGBoost (train synthetic test synthetic) r^2↑	0.318±0.02	0.316±0.007	0.389±0.011	0.198±0.0
(Performance) XGBoost (train synthetic test real) r^2↑	0.428±0.007	0.404±0.02	0.464±0.025	0.226±0.0
Delta presence ↓	7.0±3.559	5.371±4.695	3.032±1.481	1.517±0.001
k-anonymization (real data) ↑	1.0±0.0	1.0±0.0	1.0±0.0	1.0±0.0
k-anonymization (synthetic data) ↑	1.667±0.471	1.0±0.0	1.0±0.0	3.0±0.001
K-map ↑	2.0±1.414	2.333±1.247	1.667±0.471	10.0±0.0
Distinct l-diversity (real data) ↑	1.0±0.0	1.0±0.0	1.0±0.0	1.0±0.0
Distinct l-diversity (synthetic data) ↑	1.667±0.471	1.0±0.0	1.0±0.0	3.0±0.001
Identifiability score ↓	0.364±0.028	0.398±0.015	0.501±0.009	0.213±0.0
Identifiability score (one-class) ↓	0.454±0.005	0.461±0.01	0.514±0.008	0.326±0.0

Performance metrics for the different synthetic versions generated of the TCGA dataset. Arrows indicate whether a higher or lower value is preferable. Abbreviations: ADS-GAN, Anonymization through Data Synthesis using Generative Adversarial Networks; CTGAN, conditional table generative adversarial network; DDPM, denoising diffusion probabilistic models; MLP, multilayer perceptron; NHANES, National Health and Nutrition Examination Survey; PATE-GAN, Private Aggregation of Teacher Ensembles Generative Adversarial Networks; XGBoost, Extreme Gradient Boosting.

Table G. Comparison of variable distributions in the real and synthetically generated datasets (TCGA purity dataset)

Variable	Real dataset	Synthetic datasets			
		ADS-GAN	CTGAN	DDPM	PATE-GAN
**C1S**	8150 ± 14220	7864 ± 12811	7355 ± 12119	7851 ± 13855	13658 ± 23659
**CCDC69**	630 ± 1049	765 ± 1181	694 ± 1130	647 ± 1529	752 ± 1754
**CCL21**	578 ± 3560	597 ± 3734	452 ± 2915	1086 ± 11653	2502 ± 9917
**CCL22**	127 ± 396	110 ± 245	127 ± 339	172 ± 929	407 ± 1175
**CSF2RB**	312 ± 575	269 ± 433	334 ± 663	346 ± 1018	654 ± 1612
**CYTIP**	239 ± 345	308 ± 436	222 ± 281	242 ± 457	400 ± 721
**FGR**	227 ± 279	165 ± 194	208 ± 228	231 ± 368	469 ± 813
**IL7R**	118 ± 209	138 ± 225	85 ± 164	114 ± 231	125 ± 256
**POU2AF1**	296 ± 1043	270 ± 777	353 ± 995	384 ± 2302	532 ± 2078
**RHOH**	180 ± 420	203 ± 411	157 ± 351	172 ± 490	301 ± 729
**Purity**	0.6250 ± 0.2086	0.6208 ± 0.1978	0.6194 ± 0.2140	0.6354 ± 0.2092	0.5503 ± 0.2303

The set of genes presented was selected from Li et al. (2019) [3] and tumour purity was derived using the ABSOLUTE method [4].

All data are mean (± standard deviation). The distribution of variables in the original selection of data from the TCGA purity dataset (i.e., the "real" dataset) and four synthetic datasets from various methods are given. Abbreviations: ADS-GAN, Anonymization through Data Synthesis using Generative Adversarial Networks; CTGAN, conditional table generative adversarial network; DDPM, denoising diffusion probabilistic models; PATE-GAN, Private Aggregation of Teacher Ensembles Generative Adversarial Networks

.

Table H. Performance on the TCGA gene purity dataset

Method	Mean squared error (mean ± SD)
Real data	0.0208 ± 0.0008
ADS-GAN	0.0218 ± 0.0007
CTGAN	0.0258 ± 0.0009
DDPM	0.0213 ± 0.0009
PATE-GAN	0.0453 ± 0.0011

XGBoost performance predicting the tumour purity in bulk RNA samples using a previously derived prognostic gene expression signature[3,4]. Results are averaged across 5-folds for each method and reported with the corresponding standard deviation. Abbreviations: ADS-GAN, Anonymization through Data Synthesis using Generative Adversarial Networks; CTGAN, conditional table generative adversarial network; DDPM, denoising diffusion probabilistic models; PATE-GAN, Private Aggregation of Teacher Ensembles Generative Adversarial Networks

.

Table I. Ablation studies comparing SynthCraft against GPT-4o

Workflow action	SynthCraft using GPT-4o	Ablation studies		
		GPT-4o run 1	GPT-4o run 2	GPT-4o run 3
Upload data file	✓	✓	✓	✓
Exclude/keep columns	✓	✗	✗	✗
Perform exploratory data analysis	✓	✗	✓	✓
Generate descriptive statistics	✓	✗	✗	✗
Confirm ML problem type	✓	✓	✓	✓
Offer the user privacy plugins	✓	✗	✓	✗
Select synthetic data generation method	✓	✓	✓	✓
Offer augmentation	✓	✗	✗	✗
Generate synthetic data	✓	✓	✓	✓
Calculate the metrics for the generated data	✓	✓	✓	✓
Explain and compare metrics for different methods	✓	O - Only when directly asked to do this on the 3rd ask	✗	✗
Perform exploratory data analysis	✓	✓	✗	✗
Generate descriptive statistics	✓	✗	✗	✗
Plot synthetic data	✓	O - Called but when asked to do something else	✗	✗
Summarise the project	✓	✗	✗	✗
**Other issues**				
Important steps require confirmation from user	✓	✗	✗	✗
Calls correct tools in response to feedback	✓	✗	✓	✓
No useless tool calls	✓	✗	✓	✓
No code generated that fails to run	✓	O	O	O
Explains the different generators	✓	✗	✗	✗
Quality synthetic data comparison	✓	✗	✗	✗
Detailed description of the methods to choose between	✓	✗	✗	✗

Ablation studies comparing the performance of SynthCraft (using GPT-4o [API version: 2024-08-06] but with reasoning engine) by comparison with the use of GPT-4o (OpenAI) alone equipped with the same tools. This highlights that SynthCraft remains robust to different LLM versions (GPT-4o here compared to GPT-5 in the main analyses).

SUPPLEMENTARY FIGURES

Fig A. Workflow for the NHANES dataset

The following set of screenshots, illustrate the user-AI interaction when using SynthCraft.

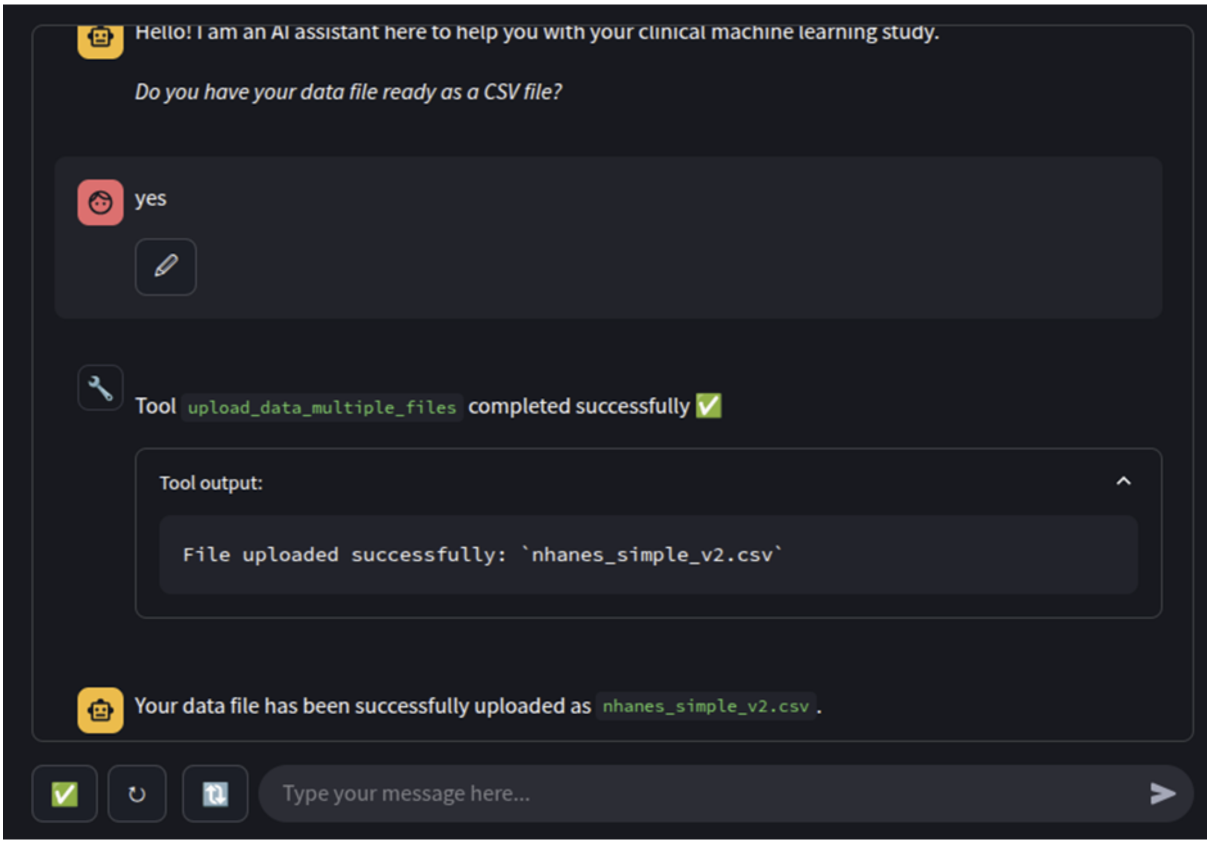

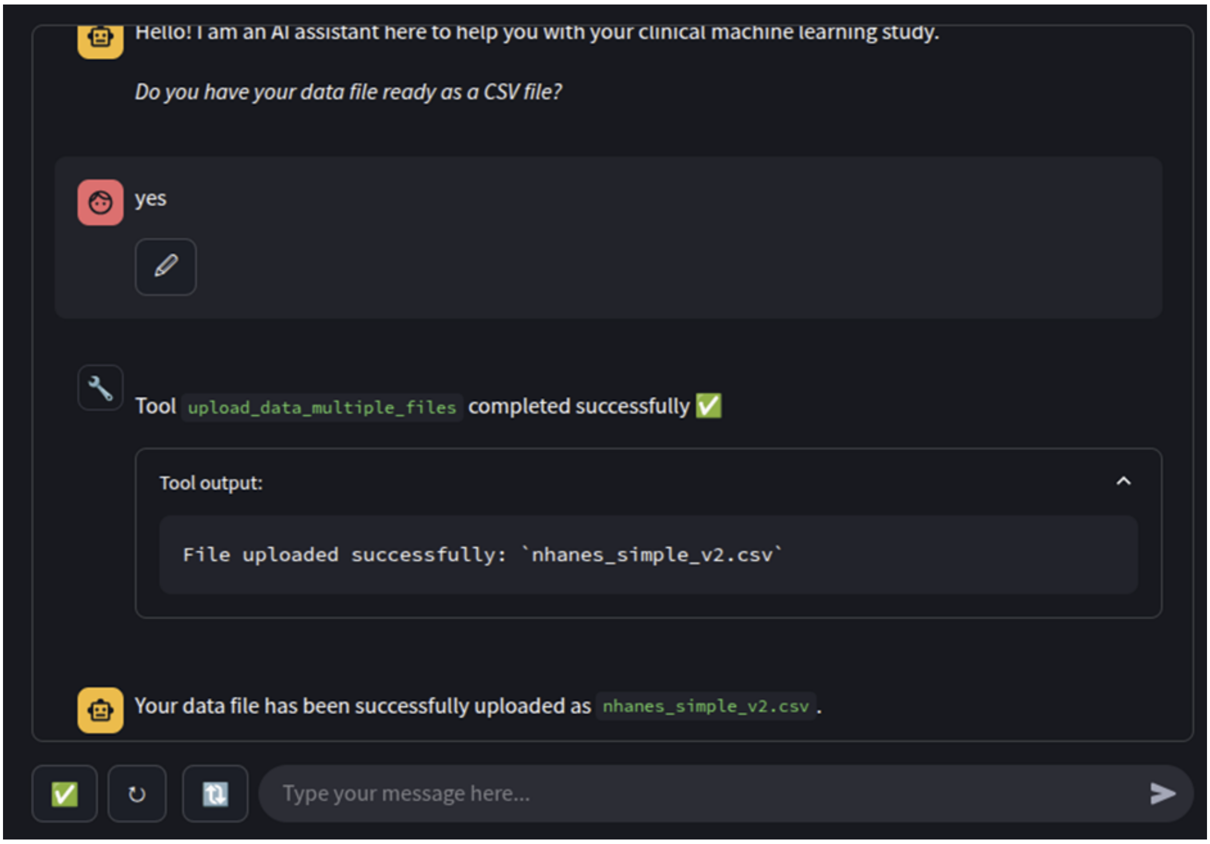
Hello! I am an AI assistant here to help you with your clinical machine learning study.
Do you have your data file ready as a CSV file?
yes
Tool upload_data_multiple_files completed successfully ✅
Tool output:
File uploaded successfully: `nhanes_simple_v2.csv`
Your data file has been successfully uploaded as nhanes_simple_v2.csv.
Type your message here...

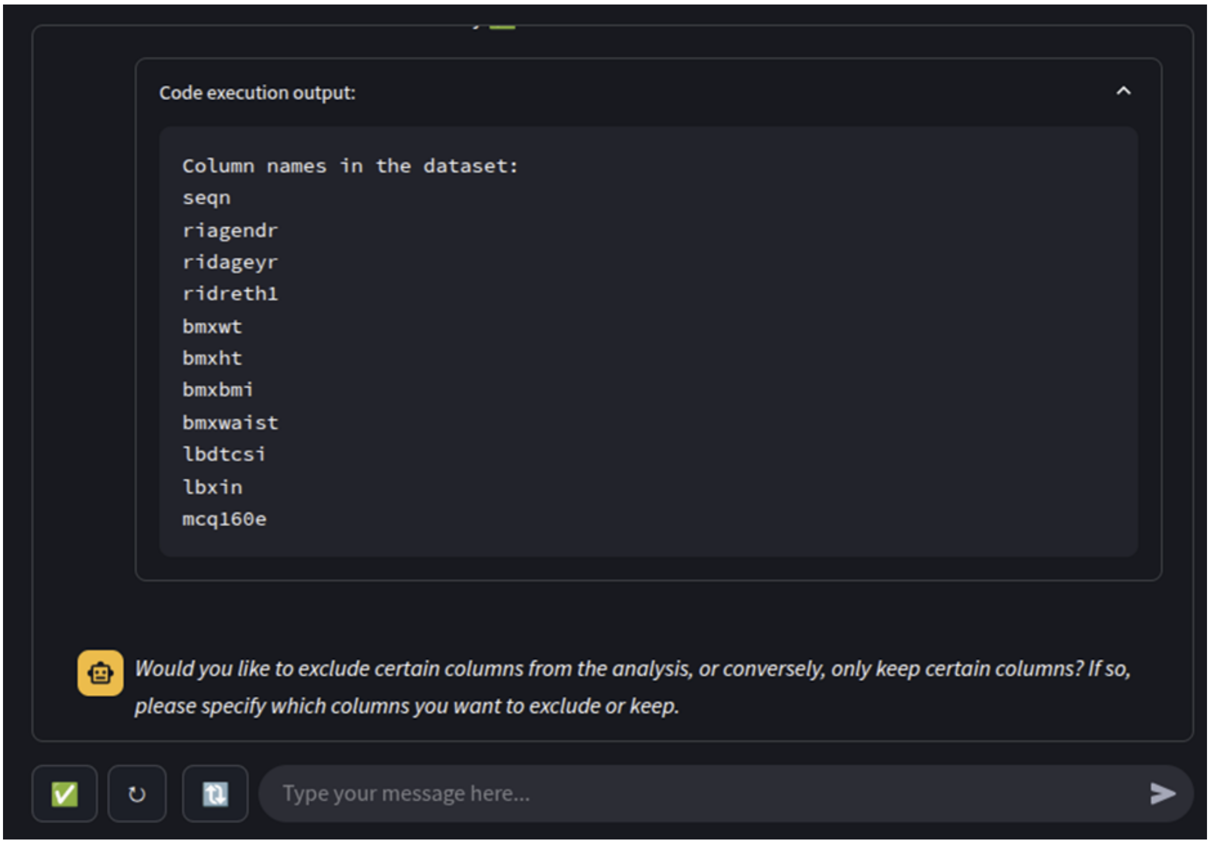
Code execution output:
Column names in the dataset:
seqn
riagendr
ridageyr
ridreth1
bmxwt
bmxht
bmxbmi
bmxwaist
lbdtcsi
lbxin
mcq160e
Would you like to exclude certain columns from the analysis, or conversely, only keep certain columns? If so, please specify which columns you want to exclude or keep.
Type your message here...

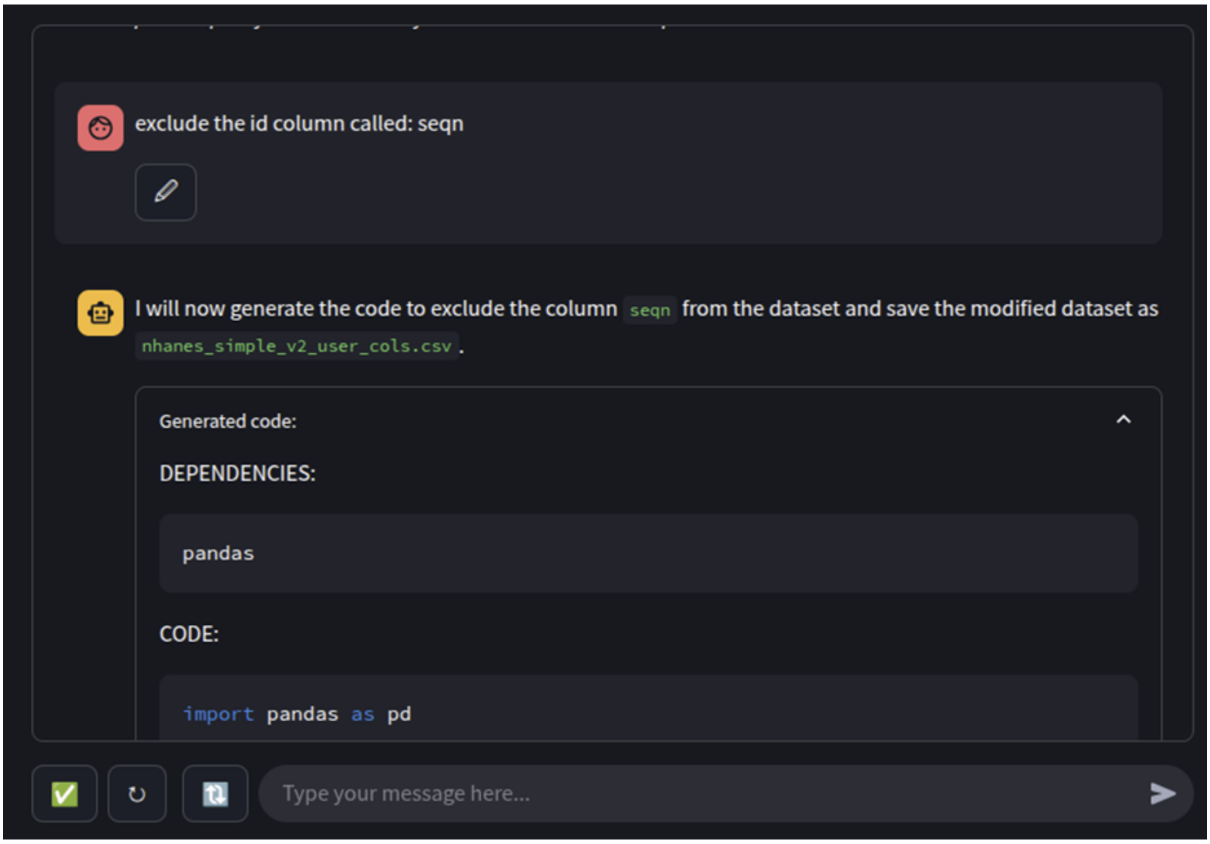
exclude the id column called: seqn
I will now generate the code to exclude the column seqn from the dataset and save the modified dataset as nhanes_simple_v2_user_cols.csv.
Generated code:
DEPENDENCIES:
pandas
CODE:
import pandas as pd
Type your message here...

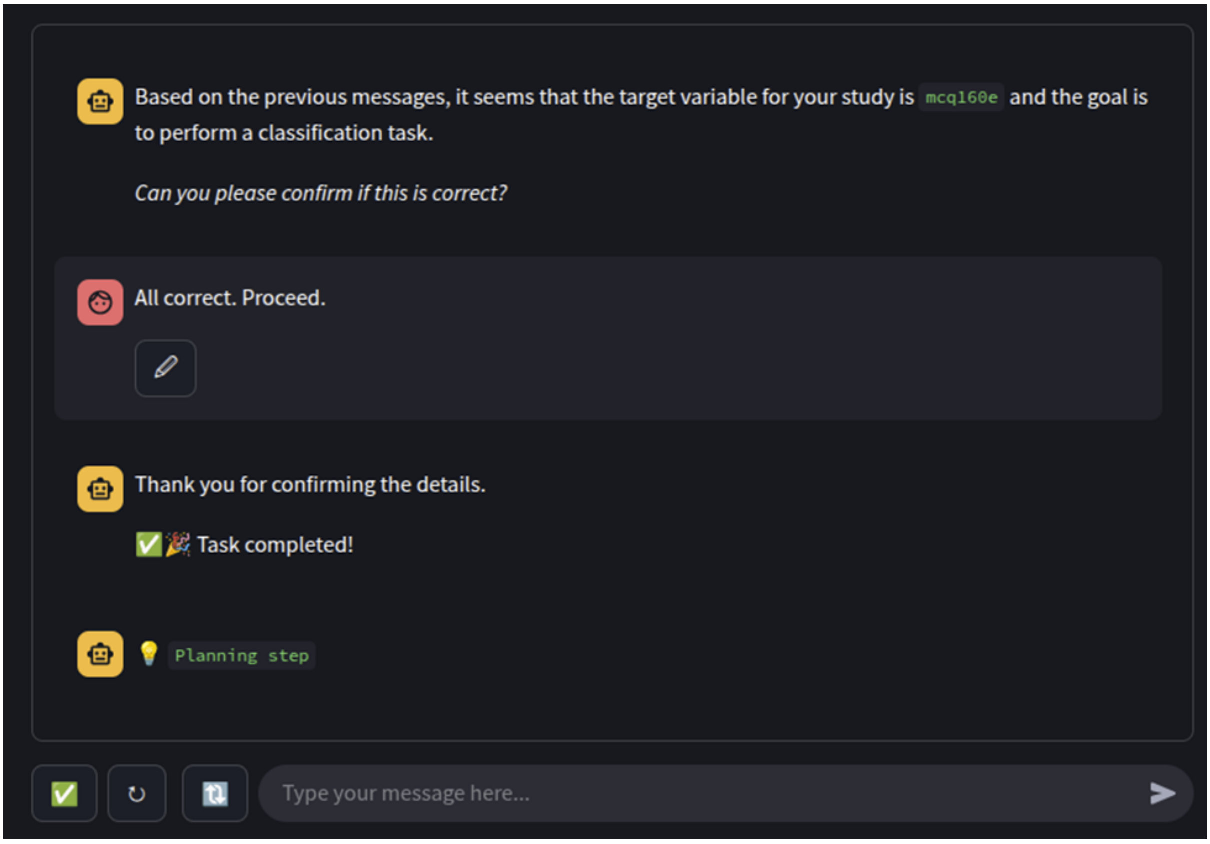
Based on the previous messages, it seems that the target variable for your study is mcq160e and the goal is to perform a classification task.
Can you please confirm if this is correct?
All correct. Proceed.
Thank you for confirming the details.
✅🎉 Task completed!
💡 Planning step
Type your message here...

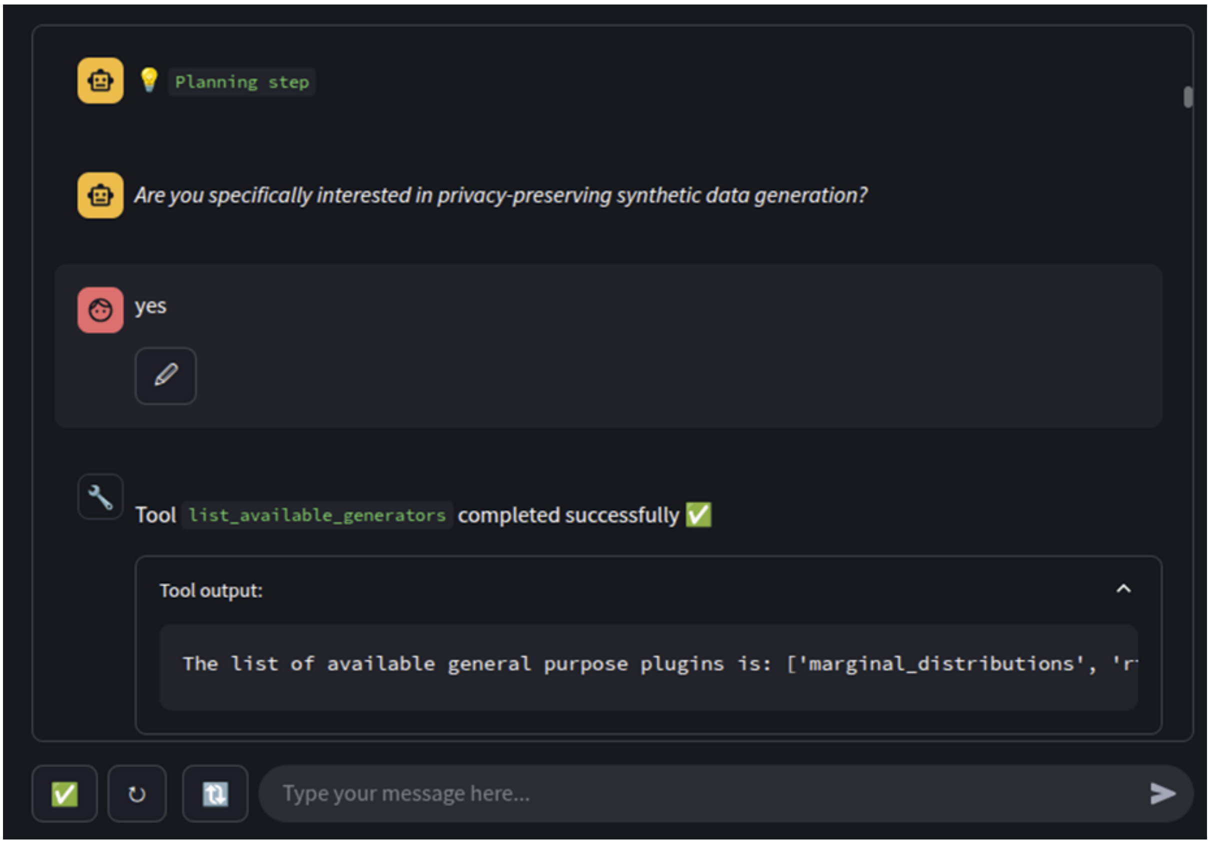
Planning step
Are you specifically interested in privacy-preserving synthetic data generation?
yes
Tool list_available_generators completed successfully
Tool output:
The list of available general purpose plugins is: ['marginal_distributions', 'r
Type your message here...

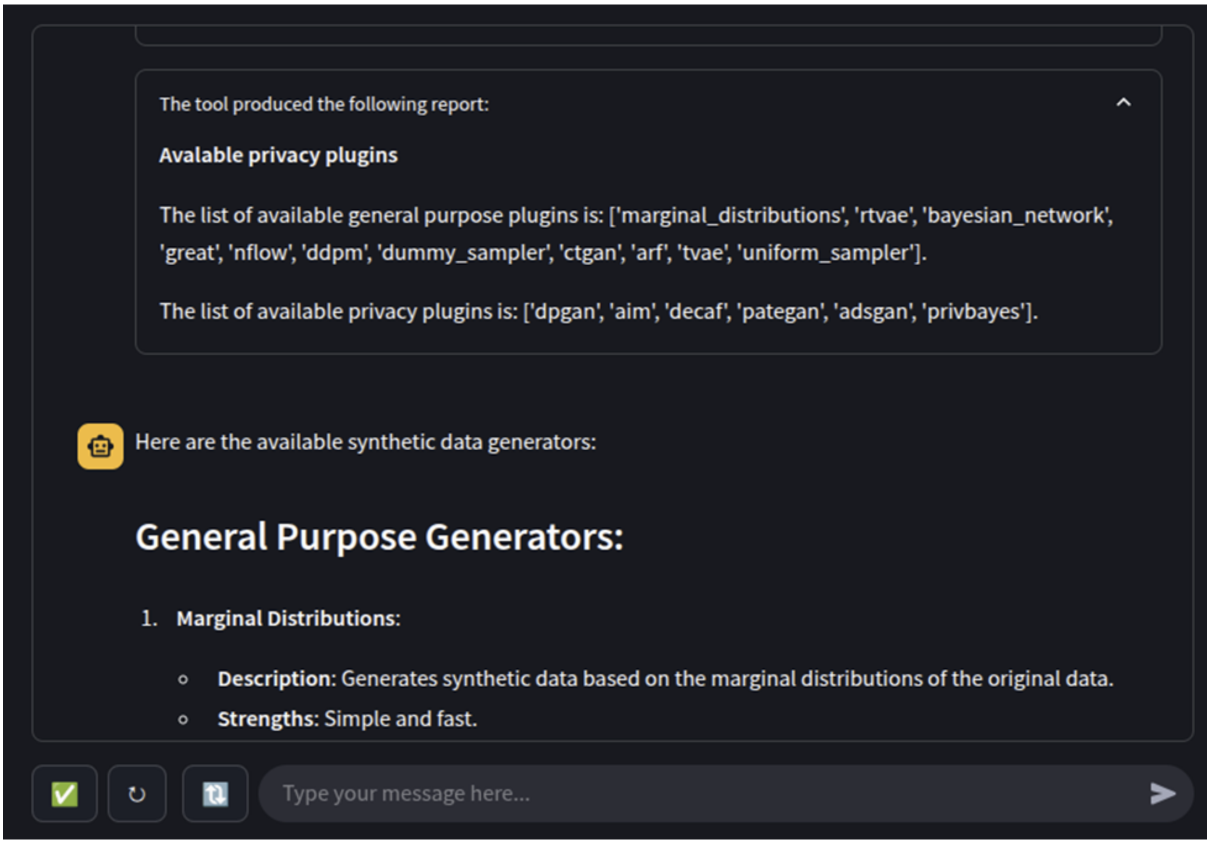
The tool produced the following report:
Avalable privacy plugins
The list of available general purpose plugins is: ['marginal_distributions', 'rtvae', 'bayesian_network', 'great', 'nflow', 'ddpm', 'dummy_sampler', 'ctgan', 'arf', 'tvae', 'uniform_sampler'].
The list of available privacy plugins is: ['dpgan', 'aim', 'decaf', 'pategan', 'adsgan', 'privbayes'].
Here are the available synthetic data generators:
General Purpose Generators:
1. Marginal Distributions:
Description: Generates synthetic data based on the marginal distributions of the original data.
Strengths: Simple and fast.
Type your message here...

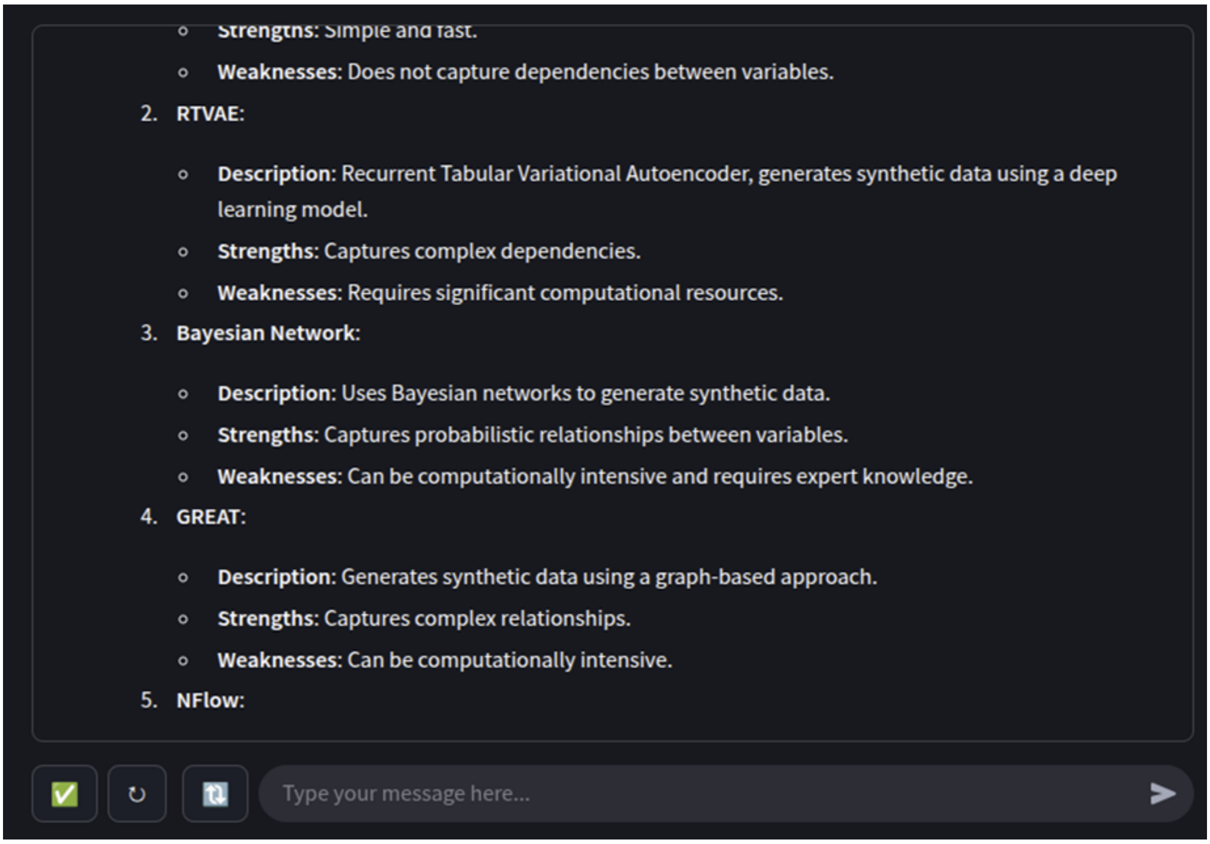

- **Strengths:** Simple and fast.
- **Weaknesses:** Does not capture dependencies between variables.

2. **RTVAE:**
   - **Description:** Recurrent Tabular Variational Autoencoder, generates synthetic data using a deep learning model.
   - **Strengths:** Captures complex dependencies.
   - **Weaknesses:** Requires significant computational resources.
3. **Bayesian Network:**
   - **Description:** Uses Bayesian networks to generate synthetic data.
   - **Strengths:** Captures probabilistic relationships between variables.
   - **Weaknesses:** Can be computationally intensive and requires expert knowledge.
4. **GREAT:**
   - **Description:** Generates synthetic data using a graph-based approach.
   - **Strengths:** Captures complex relationships.
   - **Weaknesses:** Can be computationally intensive.
5. **NFlow:**

Type your message here...

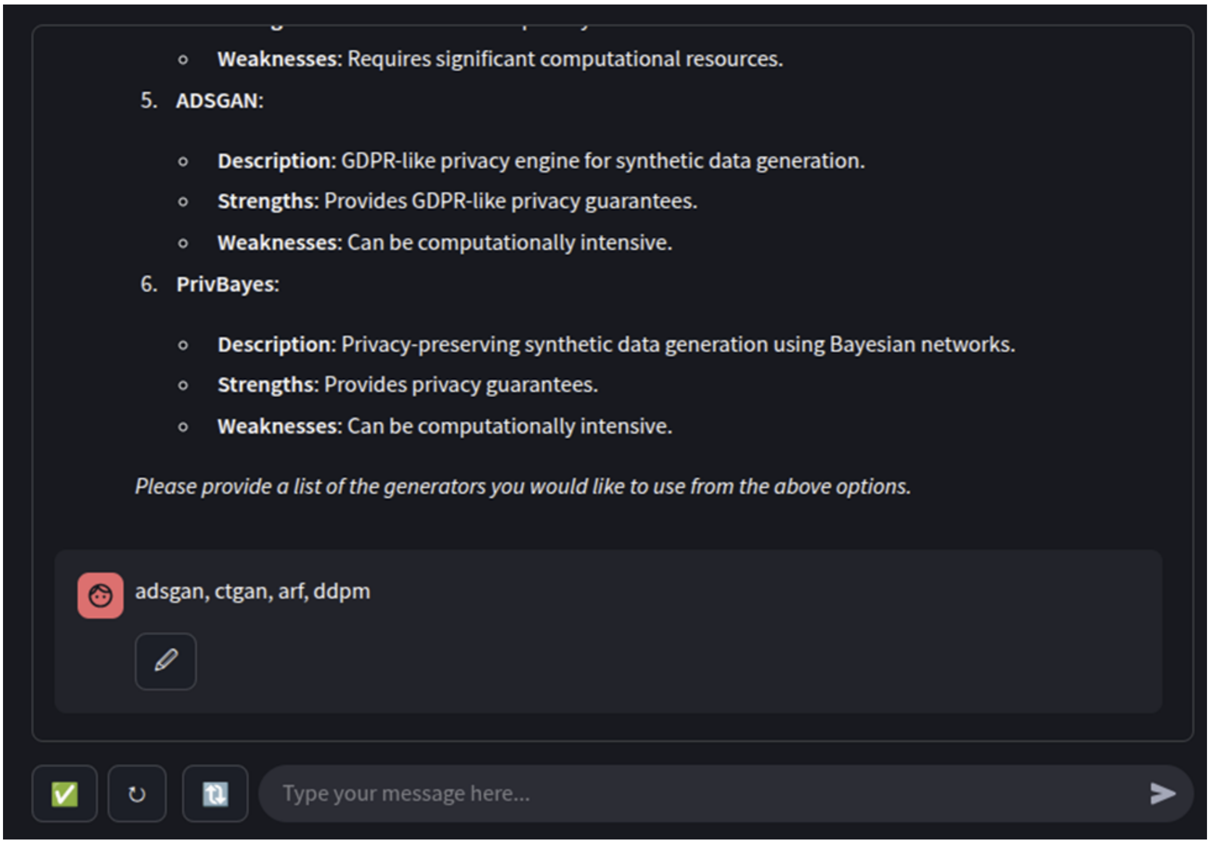

[illegible]

- **Weaknesses:** Requires significant computational resources.

5. **ADSGAN:**
   - **Description:** GDPR-like privacy engine for synthetic data generation.
   - **Strengths:** Provides GDPR-like privacy guarantees.
   - **Weaknesses:** Can be computationally intensive.
6. **PrivBayes:**
   - **Description:** Privacy-preserving synthetic data generation using Bayesian networks.
   - **Strengths:** Provides privacy guarantees.
   - **Weaknesses:** Can be computationally intensive.

Please provide a list of the generators you would like to use from the above options.

adsgan, ctgan, arf, ddpm

Type your message here...

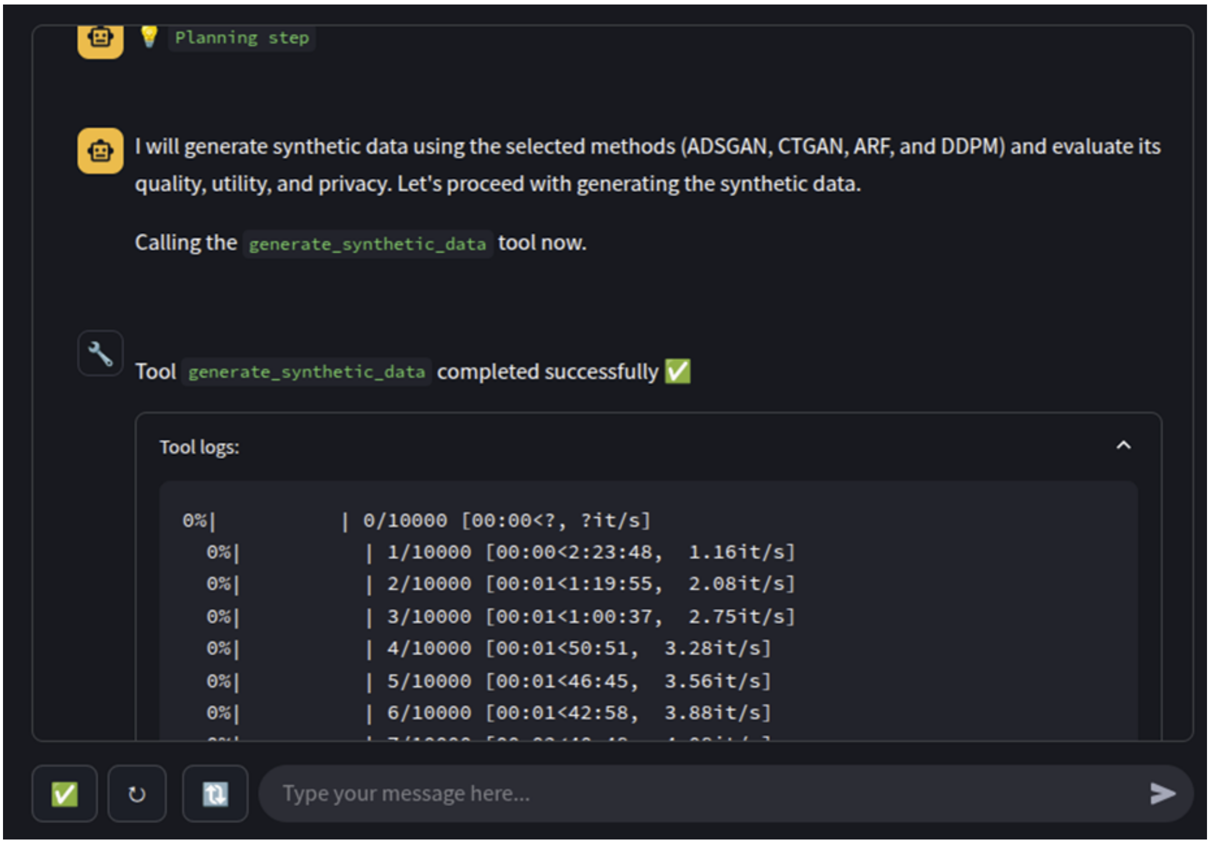
Planning step
I will generate synthetic data using the selected methods (ADSGAN, CTGAN, ARF, and DDPM) and evaluate its quality, utility, and privacy. Let's proceed with generating the synthetic data.
Calling the generate_synthetic_data tool now.
Tool generate_synthetic_data completed successfully ✅
Tool logs:
0%| | 0/10000 [00:00<?, ?it/s]
0%| | 1/10000 [00:00<2:23:48, 1.16it/s]
0%| | 2/10000 [00:01<1:19:55, 2.08it/s]
0%| | 3/10000 [00:01<1:00:37, 2.75it/s]
0%| | 4/10000 [00:01<50:51, 3.28it/s]
0%| | 5/10000 [00:01<46:45, 3.56it/s]
0%| | 6/10000 [00:01<42:58, 3.88it/s]
Type your message here...

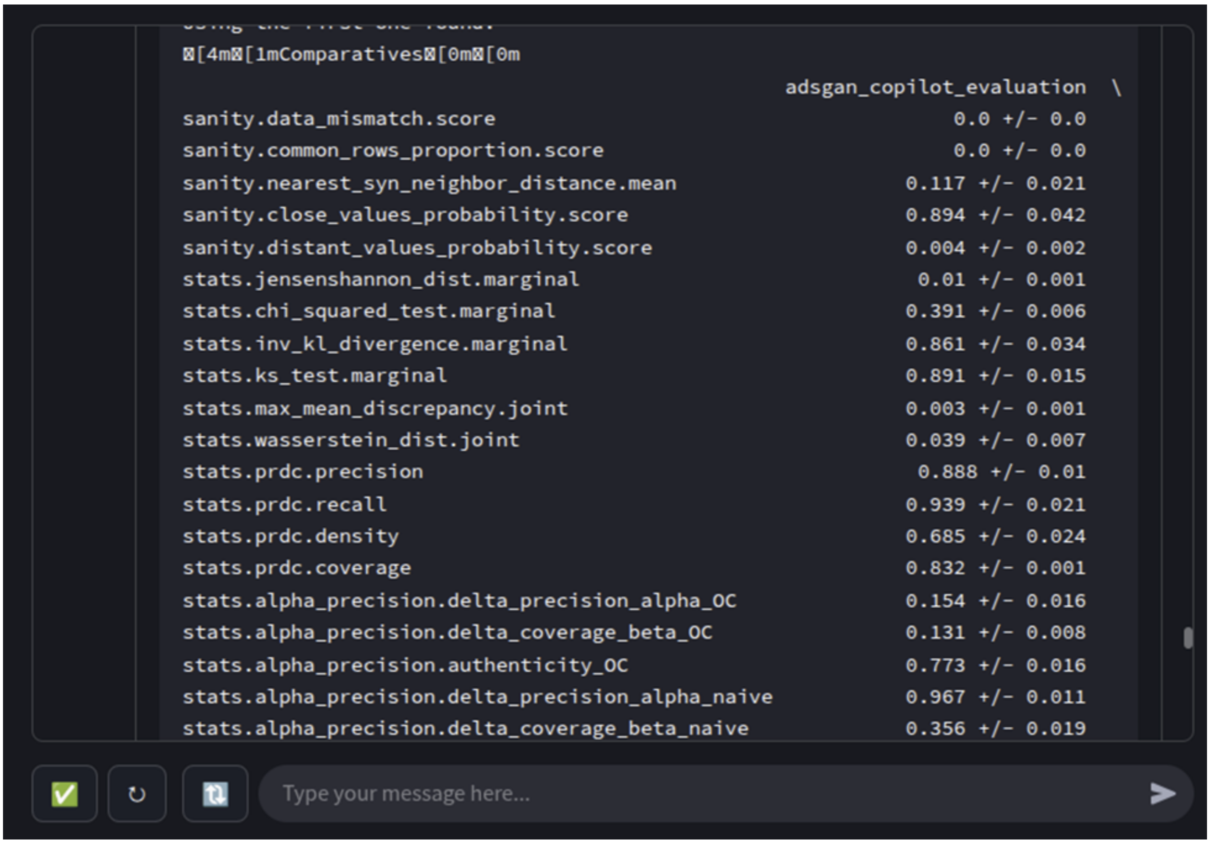
⊠[4m⊠[1mComparatives⊠[0m⊠[0m
adsgan_copilot_evaluation \
sanity.data_mismatch.score 0.0 +/- 0.0
sanity.common_rows_proportion.score 0.0 +/- 0.0
sanity.nearest_syn_neighbor_distance.mean 0.117 +/- 0.021
sanity.close_values_probability.score 0.894 +/- 0.042
sanity.distant_values_probability.score 0.004 +/- 0.002
stats.jensenshannon_dist.marginal 0.01 +/- 0.001
stats.chi_squared_test.marginal 0.391 +/- 0.006
stats.inv_kl_divergence.marginal 0.861 +/- 0.034
stats.ks_test.marginal 0.891 +/- 0.015
stats.max_mean_discrepancy.joint 0.003 +/- 0.001
stats.wasserstein_dist.joint 0.039 +/- 0.007
stats.prdc.precision 0.888 +/- 0.01
stats.prdc.recall 0.939 +/- 0.021
stats.prdc.density 0.685 +/- 0.024
stats.prdc.coverage 0.832 +/- 0.001
stats.alpha_precision.delta_precision_alpha_OC 0.154 +/- 0.016
stats.alpha_precision.delta_coverage_beta_OC 0.131 +/- 0.008
stats.alpha_precision.authenticity_OC 0.773 +/- 0.016
stats.alpha_precision.delta_precision_alpha_naive 0.967 +/- 0.011
stats.alpha_precision.delta_coverage_beta_naive 0.356 +/- 0.019
Type your message here...

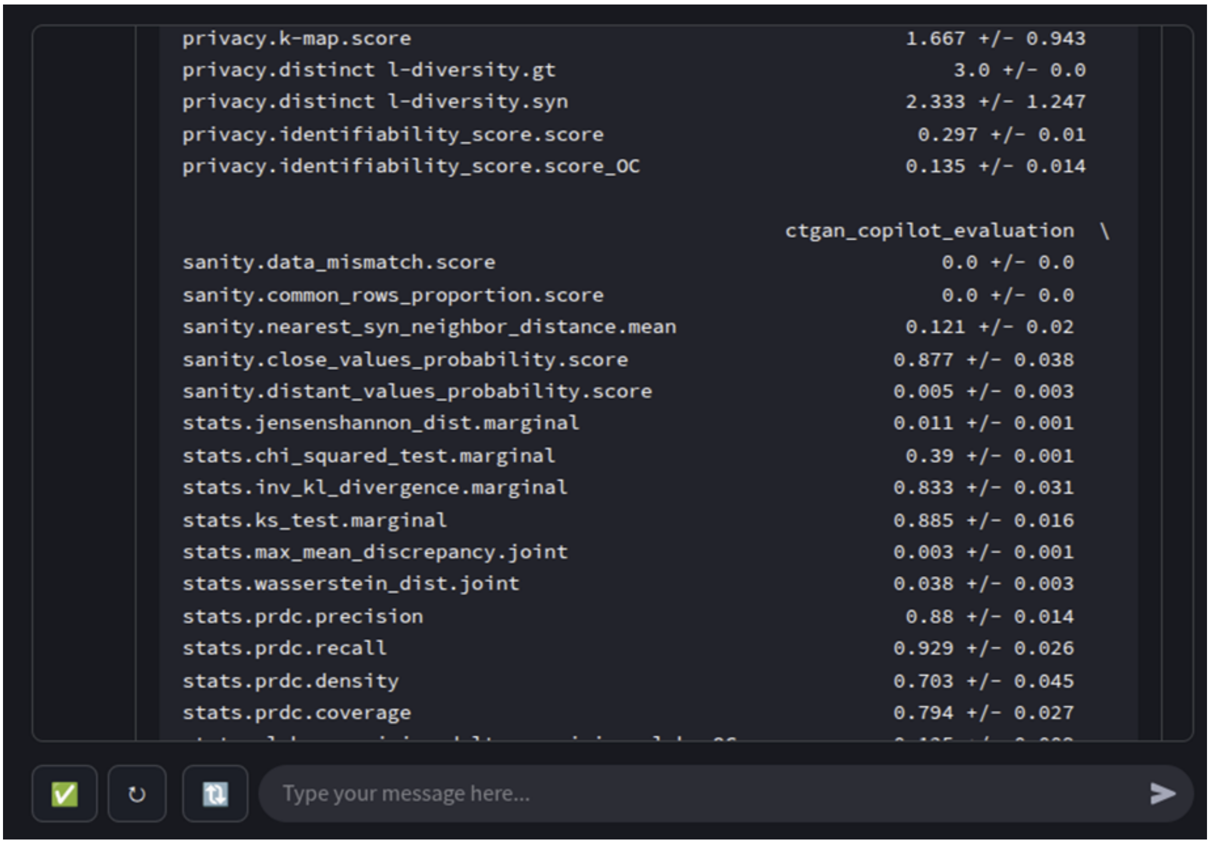
privacy.k-map.score 1.667 +/- 0.943
privacy.distinct l-diversity.gt 3.0 +/- 0.0
privacy.distinct l-diversity.syn 2.333 +/- 1.247
privacy.identifiability_score.score 0.297 +/- 0.01
privacy.identifiability_score.score_OC 0.135 +/- 0.014
ctgan_copilot_evaluation \
sanity.data_mismatch.score 0.0 +/- 0.0
sanity.common_rows_proportion.score 0.0 +/- 0.0
sanity.nearest_syn_neighbor_distance.mean 0.121 +/- 0.02
sanity.close_values_probability.score 0.877 +/- 0.038
sanity.distant_values_probability.score 0.005 +/- 0.003
stats.jensenshannon_dist.marginal 0.011 +/- 0.001
stats.chi_squared_test.marginal 0.39 +/- 0.001
stats.inv_kl_divergence.marginal 0.833 +/- 0.031
stats.ks_test.marginal 0.885 +/- 0.016
stats.max_mean_discrepancy.joint 0.003 +/- 0.001
stats.wasserstein_dist.joint 0.038 +/- 0.003
stats.prdc.precision 0.88 +/- 0.014
stats.prdc.recall 0.929 +/- 0.026
stats.prdc.density 0.703 +/- 0.045
stats.prdc.coverage 0.794 +/- 0.027
Type your message here...

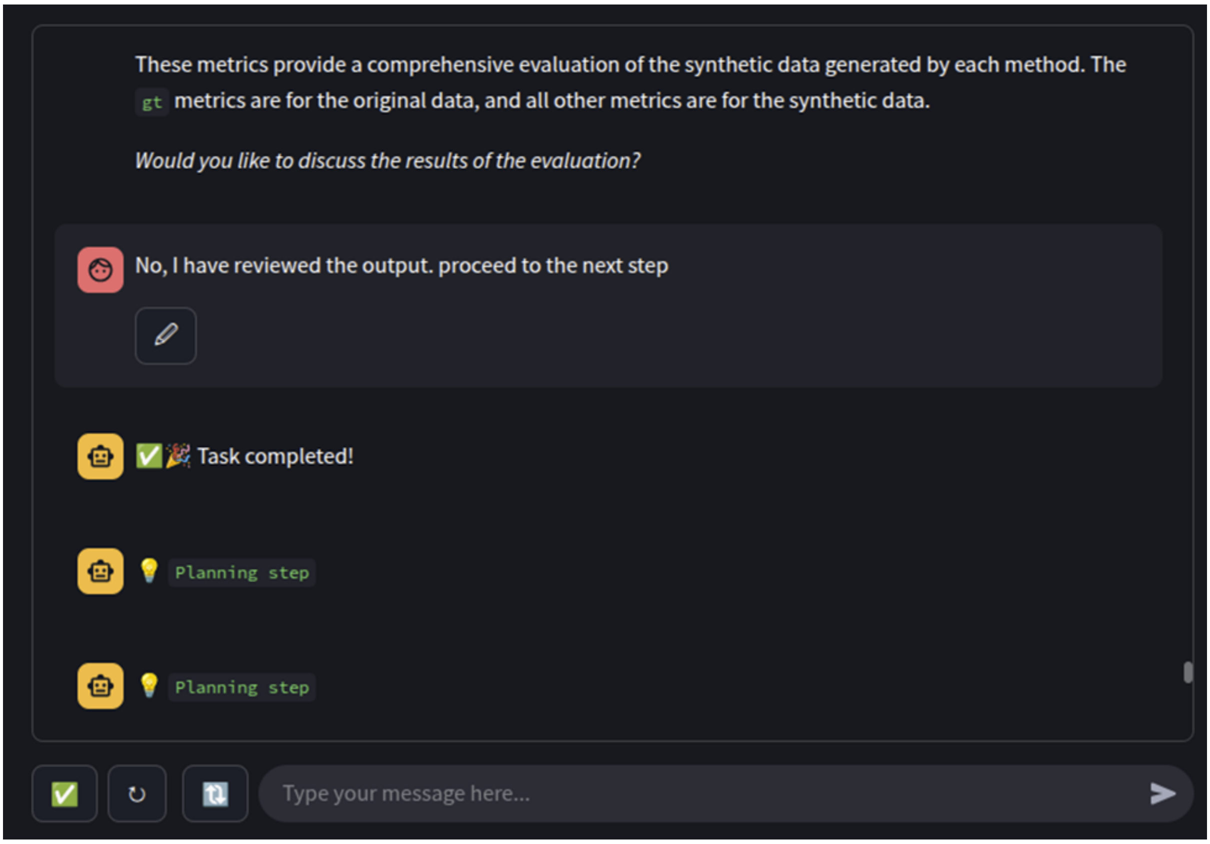
These metrics provide a comprehensive evaluation of the synthetic data generated by each method. The gt metrics are for the original data, and all other metrics are for the synthetic data.
Would you like to discuss the results of the evaluation?
No, I have reviewed the output. proceed to the next step
✅🎉 Task completed!
💡 Planning step
💡 Planning step
Type your message here...

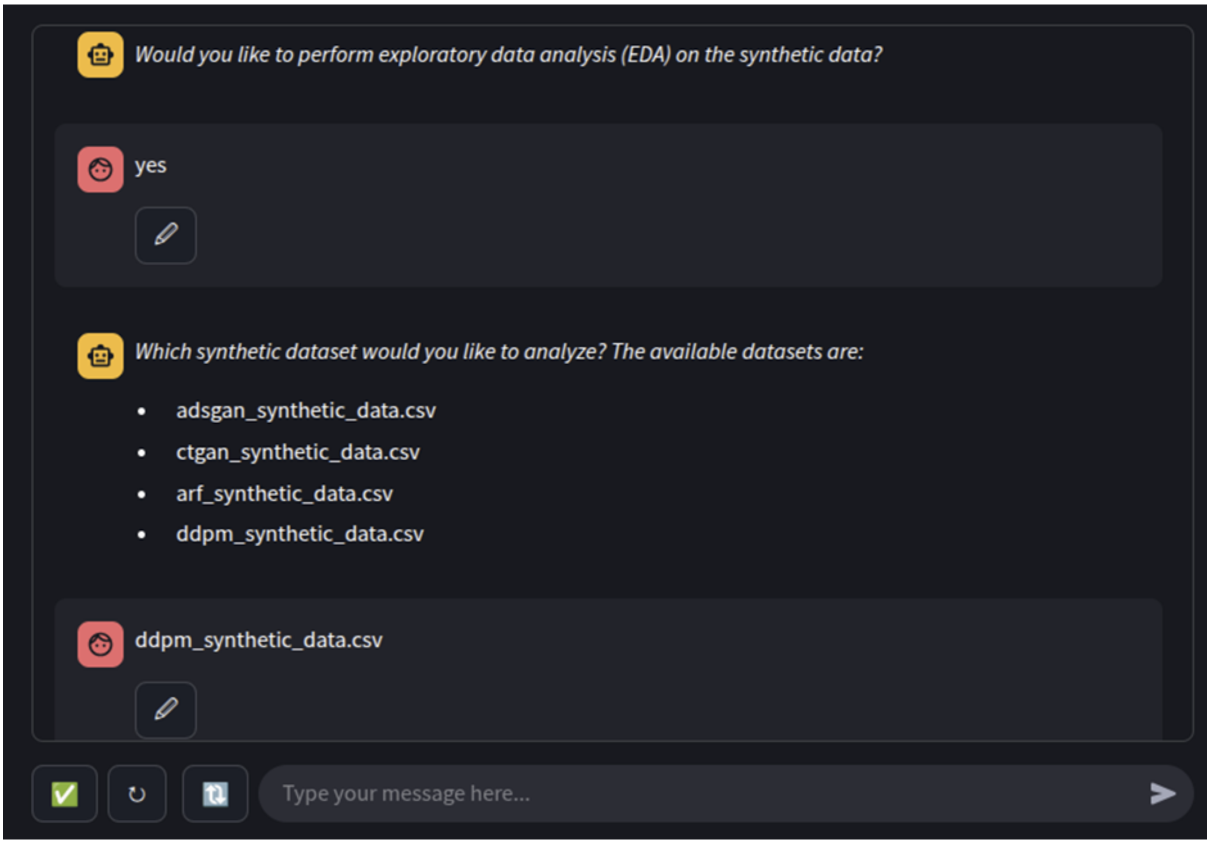
Would you like to perform exploratory data analysis (EDA) on the synthetic data?
yes
Which synthetic dataset would you like to analyze? The available datasets are:
adsgan_synthetic_data.csv
ctgan_synthetic_data.csv
arf_synthetic_data.csv
ddpm_synthetic_data.csv
ddpm_synthetic_data.csv
Type your message here...

REFERENCES

1. Alaa A, Van Breugel B, Saveliev ES, van der Schaar M. How Faithful is your Synthetic Data? Sample-level Metrics for Evaluating and Auditing Generative Models. In: Chaudhuri K, Jegelka S, Song L, Szepesvari C, Niu G, Sabato S, editors. Proceedings of the 39th International Conference on Machine Learning. PMLR; 2022. pp. 290–306. Available: https://proceedings.mlr.press/v162/alaa22a.html

2. Qian Z, Cebere B-C, van der Schaar M. Synthcity: facilitating innovative use cases of synthetic data in different data modalities. arXiv [csLG]. 2023. Available: http://arxiv.org/abs/2301.07573

3. Li Y, Umbach DM, Bingham A, Li Q-J, Zhuang Y, Li L. Putative biomarkers for predicting tumor sample purity based on gene expression data. BMC Genomics. 2019;20: 1021. doi:10.1186/s12864-019-6412-8

4. Carter SL, Cibulskis K, Helman E, McKenna A, Shen H, Zack T, et al. Absolute quantification of somatic DNA alterations in human cancer. Nat Biotechnol. 2012;30: 413–421. doi:10.1038/nbt.2203
