# Supplementary material for: SynthCraft: An AI partner for synthetic data generation to support data access and augmentation in healthcare
Source: PLOS Digit Health. 2026 Mar 9;5(3):e0001290. doi: 10.1371/journal.pdig.0001290 (PMC12970901; doi:10.1371/journal.pdig.0001290)
Supplement: Attachment — Submitted filename: synthcraft-response-to-reviewers.docx [file pdig.0001290.s002.docx]

Dear Drs Razzaghi and Celi, and peer reviewers,

Many thanks for your time and feedback - we’ve made the changes requested and would be happy to make further adjustments as needed.

We look forward to hearing from you.

With best wishes,

Tom Callender and colleagues

**Reviewer #1:** This is a great and timely contribution to the field of digital health and AI for biomedical data science. The authors introduce SynthCraft, a human-in-the-loop AI partner that leverages LLM reasoning and reinforcement learning to orchestrate synthetic data generation, evaluation, and reporting. The system lowers technical barriers for researchers, promotes transparency, and enhances reproducibility in data access and augmentation workflows.

Many thanks for your kind comments.

Suggestions for improvement:

1. Clarify LLM involvement and reproducibility:

- Specify the reproducibility of results given that GPT-4 (temperature = 0.5) introduces nondeterminism. Would different LLM versions or parameters affect workflow outcomes?
- Explicitly state how prompts and agent reasoning are logged or shared for transparency.

Thank you for this suggestion, which prompted us to update the system to GPT-5 and re-run our analyses. We hope you will agree has strengthened our conclusions.

We have now:

- Updated all analyses to use GPT-5.
- We have documented several ablation runs where we used GPT-5 with access to the same tools, but without the SynthCraft reasoning engine, to quantify the added value that our methodological approach holds over using state-of-the-art LLMs alone for biomedical research.
- We have created a new table in the Appendix (Supplementary Table S9) which compares the workflow using SynthCraft with GPT-4o alongside ablation studies of using GPT-4o as a standalone LLM. This allows readers to directly compare the fact that SynthCraft is robust to the use of these different LLM versions. The impact of changing the LLM has had no bearing on the quality or utility of the downstream synthetic data generated in our analyses. This was expected because both versions use a well-established package – Synthcity – to train synthetic data generators independently of the LLM versions.

With regards prompts and reasoning, as an open-source project the system level prompts are provided in full at <https://github.com/vanderschaarlab/SynthCraft>. For each run, the user can see both the prompts and code generated by the system. These can be directly saved. In the future, should specific guidelines be published for the generation of synthetic data (e.g. analogous to TRIPOD in prediction models), the summarisation log (shown in the Supplementary Appendix) can be updated to comply. To highlight this, we have adjusted one of the sentences in the methods to now read:

User prompts and any code generated by SynthCraft can be saved directly whilst, on completion, a structured report detailing all steps taken, decisions made, and code run is produced to support transparency and reproducibility.

2. Quantify computational efficiency and scalability:

- Include run times or resource requirements for SynthCraft workflows on both datasets to guide prospective users.

Thank you for this point. The degree of resource required will vary between use cases and datasets. With any analysis of synthetic data, the rate limiting factor will most often be the time taken to train the synthetic data generators, which will vary between datasets and use cases, but does not impact LLM resources (i.e. tokens) directly as SynthCraft performs these analyses locally using the Synthcity package.

3. Expand on limitations of privacy evaluation: While metrics like k-anonymity and authenticity are discussed, consider including a brief analysis or discussion of differential privacy guarantees across the models.

Thank you for prompting us to expand on this. We have updated the methods section to more thoroughly discuss differential privacy, protection against reidentification attacks, and the role of such mathematical guarantees in tandem with rigorous evaluation of the synthetic data generated. The relevant section now reads:

PATE-GAN, DDPM, and ADS-GAN are specifically privacy-preserving synthetic data generators. PATE-GAN uses differential privacy, a mathematical framework that ensures the inclusion or exclusion of a single data point does not significantly affect the output of data analysis [25]. We used a default privacy epsilon of 1 for these analyses based on previous work that has shown that this value can still provide strong privacy guarantees with minimal impact on the usefulness of the synthetic data [21]. As the epsilon is reduced, the privacy of the resulting synthetic data rises but with a trade-off in terms of its utility for downstream tasks such as prognostic modelling [21,26]. DDPM and ADS-GAN are specifically tailored to protect against re-identification attacks, where an adversary attempts to identify individuals within anonymised or pseudonymised datasets. CTGAN has no additional privacy-preserving framework embedded. Many generators will produce synthetic data that preserve privacy. However, generators that use differential privacy or protect against reidentification attacks provide users with specific, tuneable, mathematical guarantees over the privacy and fidelity of the resulting synthetic data. Such guarantees do not, on their own, reduce the need for rigorous evaluation of the privacy of the datasets generated.

To evaluate the quality, utility, and privacy of the generated data, we focused on several performance metrics (an exhaustive list of metrics and results are presented in Supplementary Table S2) [9,27,28]…

4. Discuss user experience and usability testing: Since SynthCraft aims to democratize access to synthetic data generation, some preliminary feedback from non-expert users (clinicians, data scientists) would strengthen the claim of accessibility.

Thank you. We have had informal user feedback from people in the United Kingdom and Switzerland from a variety of backgrounds – clinical, AI, biomedical research – with encouraging results. In addition, the tool has been showcased through both the Cambridge Centre for AI Medicine Summer School (Autumn 2025) and in the NLDL Nordic AI Winter School (Jan 2026), where more than 100 participants have been exposed to this technology with some able to experiment with it directly.

In our feedback, we have found that users have particularly valued several aspects. First, the fact that they could ask the system about the models, to clarify their respective strengths and weaknesses for their specific use case. Second, that they could continuously ask clarifying questions throughout the analysis pipeline. And, third, that they could use the system to iteratively generate appropriate synthetic data, adapting the analyses to their needs that become clearer as they progress through the workflow. In response to feedback, we are continuing to improve documentation to make it more straightforward to set up different LLMs and to use the secure Azure instances of OpenAI.

A key element of the work is that it is designed as an open-source project. We want to encourage active community engagement, allowing the software to be improved over time; we hope that the analyses presented in this paper will improve outreach and highlight the potential of the work.

**Reviewer #2:** - In Table 1, the authors have reported the units of Insulin as uU/mL. Do you mean micro-U/mL? Please use μ instead of u to avoid any confusion. the same problem is present in Table 2.

Thank you for highlighting this – we have updated the text.

- In the methods, dataset section authors have declared to include waist circumference from NHANES data set but the data was not reported in results section.

We apologise. This is an oversight – we did not include waist circumference in the models and so have removed this from the methods.

- The major innovation of this study is the integration of a LLM model with four types of generator models. The idea is simple yet effective, but the problem with LLM models is the incorrect suggestions from the generator model. the LLM may increase the bias and inaccuracy by hallucination. there is also the problem of keeping the LLM model updated. To these reasons introduction of LLM model may increase the needed computational power, increased inaccuracy, and reduce tractability in the generated data.

Thank you for this point. It is precisely to address these concerns that we developed SynthCraft. The key reason that our method is protected from any bias or inaccuracy introduced through an LLM hallucinating is that, in SynthCraft, the LLMs are not generating synthetic data – we use a well-debugged, open-source, package Synthcity for this.

The role of the LLM is limited to user interaction and it is not responsible for writing code to generate synthetic data itself. LLMs are only used within our reasoning and work agents:

- The reasoning agent plans the order of tasks and keeps the user on track while accommodating their requests and questions.
- The worker agent interacts with the user and obtains the information necessary to generate synthetic data. When the worker agent has the information needed, it hands-over the process to the non-LLM-powered-workflow of Synthcity, the independent package that runs the generation.

The only place where an LLM error could occur is if the worker agent hallucinates the parameters to pass to Synthcity. We protect against this in 2 ways: a) the worker agent is instructed to confirm the parameters with the human user before moving on, and b) the human is kept-in-the-loop and can question or revert any steps. This would trigger the reasoning agent to re-plan the last step and any hallucination can thus be caught. To elaborate on this in the paper, we have updated the methods so that the relevant paragraph with the sub-section “Overview of SynthCraft” reads:

SynthCraft is a modular LLM framework, in which a coordinating LLM agent controls worker agents with the ability to generate code and run specialised synthetic data generation tools. This framework includes several important guardrails: first, the LLMs are not responsible for writing code to generate or evaluate synthetic data, instead using Synthcity (version 0.2.12). Second, we protect against the introduction of errors at the point of generating synthetic data by ensuring the human user is asked to validate key parameters before Synthcity is invoked. More broadly, the user is kept in the loop throughout, can ask questions at any point, and can revert steps. We used GPT-5 from OpenAI (model: GPT-5; API version: “2025-08-07”; temperature: 0.5) on a secured instance of Azure as the underlying LLM in these analyses, but an alternative LLM could be simply interchanged to underpin the agents in this framework.

By contrast with LLMs alone, we show that our approach – involving a reasoning engine coupled with hard-coded pipelines and guardrails in the form of an established package for synthetic data generation – can lead to more accurate, transparent, and reproducible research. In our new analyses, we have also compared the performance of the system across two different versions of LLMs and have not found that this has impacted the quality of the synthetic data generated.

**Reviewer #3:** Clarify the core contribution
The manuscript should more clearly articulate what is methodologically novel about SynthCraft beyond integrating existing tools (Synthcity + LLMs). A concise bullet list of contributions at the end of the Introduction would help distinguish between (a) the agentic framework and (b) the empirical comparison of synthetic data generators.

Thank you for this helpful suggestion. We have now added a concise list to the last paragraph of the introduction to more clearly articulate our methodological contributions as distinct from the empirical evaluations. Specifically, we more clearly emphasise how SynthCraft brings a principled orchestration layer that ensures methodological rigour with human-in-the-loop collaboration. In the revised paper, we also demonstrate how this is something that LLMs alone cannot do (Table 1).

The broader implication is that such systems could represent a new avenue for improving the rigour, reproducibility, and accessibility of state-of-the-art ML in biomedical research. The newly added/adjusted text is below – we hope that it appropriately signals where the paper is leading.

…In developing this system, we first show the necessity of bespoke reasoning systems over using LLMs alone before comparing the performance of SynthCraft using different LLMs. We then demonstrate the capability of SynthCraft across tabular and genomic datasets for both data access and augmentation tasks.

Detail the agent / bandit reasoning module
The description of the episodic multi-armed bandit is too high-level. Please formalize the state, action space, reward function, and learning procedure, and explain how this component actually influences pipeline decisions compared with simple rule-based orchestration.

To make this clearer in the main text, we have added more detailed mathematical formulation of the bandit reasoning module, how the states, action space, and reward functions work, along with how this influences pipeline workflow. This is now available in the main text as Box 1 (below); by adding this as a box, we hope this maintains the flow of the main text for less technical readers without compromising access to this information for those who would like it.

**BOX 1: Details of the SynthCraft reasoning engine**

SynthCraft integrates a reasoning engine that guides users through the process of synthetic data generation using a transparent, structured decision framework. Inspired by the CLiMB architecture [16], this reasoning process is formalised as an episodic multi-armed bandit [15], tailored to maximise utility (output quality) while minimising interaction cost (user burden).

The reasoning engine consists of several core components [15,16]:

- A set of tasks (**episodes**) necessary to complete an analysis plan.
- **Costs** associated with specific actions that are returned as **feedback.**
- **Actions** to complete the tasks, from invoking tools to asking the user for feedback.
- A **state** that corresponds to all previous actions within the episode along with their costs.

Let $E$ be the set of episode types or subtasks in the synthetic data pipeline, including:

1. Dataset intake and characterisation
2. Analytic intent elicitation
3. Synthetic data generation with or without specific privacy guarantees
4. Evaluation strategy selection
5. Iterative refinement
6. Transparent documentation

Note, data pre-processing is currently expected to be performed before data are passed to SynthCraft.

Each episode, $\rho$, corresponds to one subtask $e_{\rho}\in E$, and consists of a sequence of actions ($a_{1}^{\rho},\ldots,a_{t}^{\rho}$) drawn from a set of Actions, $\mathcal{A}$ [15,16]. These actions either progress the task or end the episode and engage the user. We enforce a maximum number of actions per episode before automatically discussing with the user to prevent infinite iteration [16].

The next action to be taken $a_{t}^{\rho}\in\mathcal{A}$ draws on the previous sequence of actions and the feedback received from each action [15]. Feedback is attributed a cost, *c*, of 0 or 1, and can be received from [16]:

- external tools (e.g. the results from statistical analyses; synthetic data generators);
- LLM self-reflection; or,
- the user themselves (either mid-episode or at the end of the episode).

Feedback that requires the user is penalised (*c* = 1) to minimise user burden. At the end of episode, there is a terminal reward $R^{\rho}$ again of 1 – corresponding to approval of the actions taken by SynthCraft – or 0 if the user requires the episode to be performed differently.

The reasoning agent maintains a dynamic plan over the subtasks. A subtask, $e_{\rho}$, is considered complete when the associated episode receives a reward $R^{\rho}= 1$. The plan is not strictly sequential; SynthCraft can reorder subtasks based on task context and user feedback, maintaining flexibility and robustness across different use cases. The ultimate objective of SynthCraft’s reasoning system is to maximise terminal rewards by completing the analysis plan and its constituent steps efficiently with minimal user input [16]. All actions, feedback, and decisions are recorded to enable full reproducibility and auditability of the synthetic data workflow.

Evaluate SynthCraft as a system (not only generators)
Most results compare CT-GAN, ADS-GAN, DDPM, and PATE-GAN, but do not quantify the benefit of SynthCraft itself. Please add an evaluation comparing SynthCraft to (i) manual use of Synthcity or (ii) a simple non-bandit LLM agent, in terms of time, coverage of analyses, or error rate.

Many thanks for this suggestion, which has improved the clarity of the paper. We have:

- Re-run all the analyses having updated SynthCraft to use GPT-5 as this has been released since we initially developed the system.
- Compared SynthCraft against non-bandit LLM agents; this has allowed us to improve the clarity of our arguments around the added value of the reasoning engine and system behind SynthCraft.

We document this now in the main text with a new paragraph in the methods to describe the ablation studies as well as a new section in the results “Comparison of SynthCraft against standalone LLMs”. The new results sub-section has the following text, accompanied by a new Table 1 with the ablation studies evaluations:

SynthCraft used chain-of-thought reasoning to adapt its workflow to generate high-quality synthetic versions of both a tabular epidemiological dataset – NHANES – and a genomic dataset – TCGA (Fig 1). A complete example of this workflow can be found in Supplementary Fig S1.

To assess the value of SynthCraft over the use of state-of-the-art LLMs (GPT-5), we performed three ablation studies with the NHANES dataset. In each study, we used an instance of GPT-5 equipped with access to the same tools as SynthCraft so that we could isolate the advantages of the reasoning engine in orchestrating an end-to-end research workflow. In none of the ablation studies was GPT-5 alone able to complete the analyses (Table 1). Different error types occurred in each study, ranging from ignoring the Synthcity tool and trying to write its own code to generate synthetic data (i.e. bypassing established methods), through attempting to generate synthetic data using all possible generators available in the Synthcity package and overwhelming the compute available, to failing to evaluate the synthetic data. Importantly, in none of the ablation studies did GPT-5 involve the human user at critical stages in the workflow, which could lead to errors and inappropriate analyses.

Add an ablation or user-in-the-loop study
To support the “AI partner” claim, a small user study or ablation (e.g., with/without bandit, with/without agents) would be very valuable. Even a pilot study with a few analysts showing reduced effort or improved completeness of analyses would strengthen the practical impact.

We discuss the ablation studies and system updates in our response to the previous suggestion.

We haven’t performed a formal user study, although we have extensively discussed and tested the system with multiple people from different professional backgrounds with varying levels of coding proficiency. This will be the subject of future work. We have also showcased SynthCraft in lectures at ETH Zürich and the University of Amsterdam Medical Centers as well as the Cambridge Centre for AI in Medicine summer school and NLDL Nordic Winter School, where many participants had hands-on experience with the tools, with positive feedback. Further, as an open-source project, we expect that the tool will evolve with ongoing community engagement and feedback.

Improve methodological transparency and reproducibility
Please provide more implementation details: exact Synthcity and library versions, LLM model name, temperature, max tokens, prompt templates, and number of tool calls per episode. Consider placing full prompts and configuration files in the Supplementary Material or a public repository.

We have updated the main manuscript text with the version of SynthCity (version 0.2.12) within the Methods sub-section “Overview of SynthCraft”. The relevant sentences for the LLMs now reads as:

We used GPT-5 from OpenAI (model: GPT-5; API version: “2025-08-07”; temperature: 0.5) on a secured instance of Azure as the underlying LLM in these analyses, but an alternative LLM could be simply interchanged to underpin the agents in this framework.

Tokens and tool calls will vary depending on the use case and dataset. The full prompts and configuration files for SynthCraft are fully open-source, available at <https://github.com/vanderschaarlab/SynthCraft> (link provided in the data availability section). As SynthCraft is designed for community involvement and improvement, we feel this is the ideal location for prompts, the history of which will be recorded through open version control.

Clarify dataset preprocessing and splits
For both NHANES and TCGA, the preprocessing steps (feature selection, normalization, handling of missing data, train/test splits) should be described more precisely. This includes how outcome variables are defined, how ethnic groups or tumour purity labels are handled, and whether any information leakage might occur.

We’ve updated the statistical analysis section to more thoroughly describe highlight these steps. For these analyses, we did not perform specific feature selection when building prediction models, using the extracted variables from NHANES and relevant variables from TCGA, in the latter case so that we could recapitulate the ABSOLUTE algorithm. We have now clarified the handling of missing data and how we trained/tested models developed on synthetic data. In keeping with best practice, we trained models on synthetic data and tested them on the original real data. In the case of NHANES, because we were interested in differences in performance across sub-groups where there were sometimes relatively few outcomes of interest, we used bootstrapped confidence intervals to ensure all real data could be used rather than split sampling.

We have adjusted the relevant paragraphs so that they now read:

TCGA contains multi-omic data on tumour tissue and matched normal tissues from more than 11,000 patients alongside clinical information. For this study, we included data on tumour purity in bulk RNA sequencing (using the Illumina HiSeq 2000 platform), as determined from the ABSOLUTE algorithm [19] and a previously derived gene signature [20]. We included all tumour samples for which there were no missing values in gene expression, resulting in a dataset of 9,678 samples.
…
For the real and synthetic NHANES databases, we modelled the relationship between self-reported myocardial infarction using logistic regression and including all other variables in the dataset as covariables. We obtained odds ratios (OR) and 95% confidence intervals (CI) from these models. We used ethnicity categorisations as provided by NHANES. We used a complete case analysis. We subsequently built prediction models, analysing discriminative performance (area under the receiver operating curve; AUC) in aggregate and across sub-groups using bootstrapped (1,000 runs) confidence intervals. We present results from models that were trained on synthetic data and tested on the real dataset. For the TCGA dataset, we calculated the mean square error (MSE) of the predicted purity. We used a 5-fold cross-validation with 80% training and 20% testing data in each fold reporting the mean error across the folds alongside the standard deviation.

Deepen the analysis of privacy–utility trade-offs
The comparison of PATE-GAN with non-DP generators is interesting but somewhat superficial. Please expand the discussion on how ε was chosen, what ε≈1 implies in terms of privacy strength, and how utility degrades as privacy is strengthened. A small sensitivity analysis over ε would be very informative if feasible.

We have now expanded the methods section to explicitly describe why the epsilon was selected and to highlight your point that there is a privacy/utility trade-off. This becomes apparent in the main results where the performance of PATE-GAN is in some cases lower than other generators, but its privacy metrics substantially higher. The updated text in the methods now reads:

PATE-GAN, DDPM, and ADS-GAN are specifically privacy-preserving synthetic data generators. PATE-GAN uses differential privacy, a mathematical framework that ensures the inclusion or exclusion of a single data point does not significantly affect the output of data analysis [25]. We used a default privacy epsilon of 1 for these analyses based on previous work that has shown that this value can still provide strong privacy guarantees with minimal impact on the usefulness of the synthetic data [21]. As the epsilon is reduced, the privacy of the resulting synthetic data rises but with a trade-off in terms of its utility for downstream tasks such as prognostic modelling [21,26]. DDPM and ADS-GAN are specifically tailored to protect against re-identification attacks, where an adversary attempts to identify individuals within anonymised or pseudonymised datasets. CTGAN has no additional privacy-preserving framework embedded. Many generators will produce synthetic data that preserve privacy. However, generators that use differential privacy or protect against reidentification attacks provide users with specific, tuneable, mathematical guarantees over the privacy and fidelity of the resulting synthetic data. Such guarantees do not, on their own, reduce the need for rigorous evaluation of the privacy of the datasets generated.

To evaluate the quality, utility, and privacy of the generated data, we focused on several performance metrics (an exhaustive list of metrics and results are presented in Supplementary Table S2) [9,27,28]…

We haven’t expanded the analysis to different epsilons as we feel this would further increase the complexity of the paper without changing the methodological advance that SynthCraft provides nor necessarily providing new findings over and above those that have been published in Jordan et al. 2019 or Ganev et al. 2022.

Expand fairness and subgroup analyses in NHANES
The ethnicity-based augmentation experiment is promising, but the evaluation currently focuses mainly on overall AUC. Please report subgroup metrics (e.g., AUC or calibration per ethnic group, equal opportunity gaps) to show whether augmentation helps or harms fairness across groups.

Thank you, we have now added two new supplementary tables detailing the AUCs across different categorical sub-groups (Supplementary Tables 4 and 5) with and without augmentation. We have also redesigned Figure 2 to make the AUC panels easier to read.

We document these findings in the text in the following paragraph:

“… However, training predictive models of self-reported myocardial infarction on these augmented datasets did not improve either overall or sex- or ethnicity-specific discriminative performance (Fig 2 and Supplementary Table S4-5). Indeed, augmentation with synthetic data across all synthetic data generators led to reductions in the overall AUC and discriminative performance by subgroup by comparison with using the real data alone. Because the performance of models built on the original data were both high (AUC > 0.8) and consistent across subgroups, these findings are not unexpected. They do highlight the need for iterative testing of synthetic data both across generators and use cases, and that synthetic data can balance representation across groups, but that does not necessarily remove bias or fairness [29].”

Clarify LLM data exposure and deployment constraints
The manuscript claims that the LLM “never sees the raw data,” but this is not fully demonstrated. Please include concrete examples of prompts, explain how data are abstracted into code/text safely, and discuss safeguards to prevent users from accidentally pasting identifiable data into the LLM interface (especially in clinical deployments).

Thank you, this is an important issue. As mentioned in the response to a point above, all the prompts used by the system are freely available here: <https://github.com/vanderschaarlab/SynthCraft>.

As with any LLM-based system, the output from a tool call – for example, a descriptive analysis – could be visible to the LLM. However, the raw data would not be unless the user requests this to be printed in the output. At present, we make the risks clear to users with disclaimers. In future work, more detailed safeguards could be incorporated. However, no safeguard will ever be perfect, such that a more crucial element of the overall safety is the use of healthcare compliant versions of LLMs. To this end, we explicitly recommend their use:

“Controlled instances of LLMs are available from cloud services that are compliant with healthcare privacy regulations and should be used to add security to the system; we provide instructions on how to set up SynthCraft with this feature built in.”

Within SynthCraft’s user documentation, we have a section on Data Privacy (<https://github.com/vanderschaarlab/SynthCraft?tab=readme-ov-file>) with a “Must-read: Data Privacy documentation” page which further outlines data protection legislation and what happens in terms of data transfer when using proprietary LLMs: <https://github.com/vanderschaarlab/SynthCraft/blob/main/docs/dataprivacy.md>

Further, a user could adopt an open-source/open-weight LLM in place of a proprietary system, allowing SynthCraft to be deployed entirely within an institution’s firewalls for further protections.

Strengthen the limitations and future work section
The limitations section could be more explicit. For example: reliance on two datasets, absence of a formal usability study, dependence on proprietary LLMs, and the lack of automated hallucination detection or safety filters. Also consider outlining concrete future directions (e.g., open-weight models, more domains, better safety checks).

Thank you for these suggestions. We have strengthened the limitations/future work section to incorporate these, such that the paragraph now reads:

SynthCraft has some limitations. We found that the framework can require prompting to continue to the next stage of analyses, becoming focussed on the specific task at hand. With future research into training agent-based workflows and the ongoing improvement in underlying LLMs, we expect this limitation to be less prevalent in the future. Although the underlying LLM does not have access inherently to the data, this safeguard could be bypassed by user prompting. Controlled instances of LLMs are available from cloud services that are compliant with healthcare privacy regulations and should be used to add security to the system; we provide instructions on how to set up SynthCraft with this feature built-in. SynthCraft is also capable of being used with open-weight or open-source LLMs, providing choice over how the system is deployed and allowing for more granular privacy controls. In the development of SynthCraft we discussed the prototype with individuals from different professional backgrounds and levels of programming proficiency but have not conducted a formal user study. This will be the subject of future work. Furthermore, as an open-source project, we encourage users to suggest improvements and contribute to the development of the software. In our analyses, we demonstrate the system with two different datasets (epidemiological and genetic). Future work could test how the system performs across a broader range of datasets, including of different scales.

Improve figure and table clarity
Some figures and tables would benefit from clearer legends and more self-contained captions. Please ensure that all axes, groups, and generators are clearly labeled, use marker shapes or line styles that are robust to colour-blindness, and state in each caption what the main takeaway is.

Thank you for this suggestion which will make the figures easier to read on their own. We have added a main takeaway for the two relevant figures; updated captions for Figures 2 and 3 are shown below.

We have standardised marker/line colours to use those from Bang Wong’s Nature Methods paper on colours optimised for those with colour-blindness: <https://www.nature.com/articles/nmeth.1618>. We have also standardised the order in which synthetic data generators are presented in the Tables and Figures for consistency.

**Fig 2:** Discriminative performance (AUC) overall and by sub-group of logistic regression models trained on the original (NHANES) dataset, purely synthetic datasets (circles), or original dataset with augmentation (squares) when tested on the original real dataset. The discriminative performance of models trained purely on synthetic data can rival a model trained on real data, with the quality of models varying by synthetic dataset. Augmentation of the real dataset did not improve performance relative to using the real dataset for model development without augmentation. Abbreviations: ADSGAN, Anonymization through Data Synthesis using Generative Adversarial Networks; CT-GAN, conditional table generative adversarial network; DDPM, denoising diffusion probabilistic models; NHANES, National Health and Nutrition Examination Survey; PATE-GAN, Private Aggregation of Teacher Ensembles Generative Adversarial Networks; AUC, area under the curve.

**Fig 3:** Scatter plots illustrate the correlation between actual and predicted tumour purity for the real dataset and four synthetic datasets. Correlations between gene expression levels and estimates of tumour purity were broadly similar across synthetic and real datasets, but the mean squared error of an XGBoost regression model predicting tumour purity was noticeably different when using synthetic data generated with PATE-GAN. Abbreviations: ADSGAN, Anonymization through Data Synthesis using Generative Adversarial Networks; CT-GAN, conditional table generative adversarial network; DDPM, denoising diffusion probabilistic models; NHANES, National Health and Nutrition Examination Survey; PATE-GAN, Private Aggregation of Teacher Ensembles Generative Adversarial Networks; MSE, mean squared error.

Language, style, and consistency edits
There are minor grammatical issues and inconsistent terminology (e.g., variations in the naming of generators such as PATE-GAN / PATEGAN). A careful language pass to fix grammar, streamline long sentences, and standardize terminology will improve readability and professionalism.

Thank you for spotting this – we have adjusted PATE-GAN throughout and have reviewed for grammar, sentence structure, and terminology.
